# Structure-function analysis of CYP719As involved in methylenedioxy bridge-formation in the biosynthesis of benzylisoquinoline alkaloids and its de novo production

**DOI:** 10.1186/s12934-023-02024-2

**Published:** 2023-02-03

**Authors:** Xiuyu Liu, Xiang Jiao, Yatian Cheng, Ying Ma, Junling Bu, Baolong Jin, Qishuang Li, Zhimin Hu, Jinfu Tang, Changjiangsheng Lai, Jian Wang, Guanghong Cui, Yun Chen, Juan Guo, Luqi Huang

**Affiliations:** 1 State Key Laboratory of Dao-Di Herbs, National Resource Center for Chinese Materia Medica, China Academy of Chinese Medical Sciences, No.16 Neinanxiaojie, Dongzhimen, Beijing, 100700 China; 2 School of Pharmaceutical Sciences, Henan University of Chinese Medicine, No. 156 Jinshuidong Road, Zhengzhou, 450046 China; 3 Department of Biology and Biological Engineering, Chalmers University of Technology, Kemivägen 10, 41296 Gothenburg, Sweden

**Keywords:** CyCYP719As, Benzylisoquinoline alkaloids (BIAs), *Corydalis yanhusuo*, Methylenedioxy bridge-formation, Regiospecificity, Synthetic biology

## Abstract

Benzylisoquinoline alkaloids (BIAs) are a type of secondary metabolite with clinical application value. (*S*)-stylopine is a special BIA which contains methylenedioxy bridge structures. CYP719As could catalyze the methylenedioxy bridge-formation on the A or D rings of protoberberine alkaloids, while displaying significant substrate regiospecificity. To explore the substrate preference of CYP719As, we cloned and identified five CyCYP719A candidates from *Corydalis yanhusuo*. Two CyCYP719As (CyCYP719A39 and CyCYP719A42) with high catalytic efficiency for the methylenedioxy bridge-formation on the D or A rings were characterized, respectively. The residues (Leu 294 for CyCYP719A42 and Asp 289 for CyCYP719A39) were identified as the key to controlling the regioselectivity of CYP719As affecting the methylenedioxy bridge-formation on the A or D rings by homology modeling and mutation analysis. Furthermore, for de novo production of BIAs, CyCYP719A39, CyCYP719A42, and their mutants were introduced into the (*S*)-scoulerine-producing yeast to produce 32 mg/L (*S*)-stylopine. These results lay a foundation for understanding the structure-function relationship of CYP719A-mediated methylenedioxy bridge-formation and provide yeast strains for the BIAs production by synthetic biology.

**Supplementary Information:**

The online version contains supplementary material available at 10.1186/s12934-023-02024-2.

## Background

Benzylisoquinoline alkaloids (BIAs) represent one of the most vital plant natural products in drug discovery, such as codeine, morphine, and berberine. The dry bulb of *Corydalis yanhusuo* (named Yanhusuo) is a common traditional Chinese medicine that has been employed as an analgesic for thousands of years [1, 2]. It has been found to have anti-inflammatory, antitumor, antifibrotic, and cell-protective properties, and could alleviate pain and promote blood circulation [3]. *C. yanhusuo* is abundant in BIAs, including tetrahydroprotoberberines, quaternary protoberberines, aporphines, protopines, and benzophenanthridines [4]. (*S*)-stylopine, a protoberberine alkaloid, was identified as a promising compound due to its superior anti-inflammatory effect [5]. Synthetic biology production of BIAs has been recognized as a promising strategy to obtain adequate compounds for further investigation. However, this approach relies on the analysis of BIAs biosynthetic pathways and efficient genetic elements or pathway optimization for strain construction.

Cytochrome P450s (CYP450s) are critical post-modification enzymes which are prevalent in plant secondary metabolic pathways [6–8] and contribute to expanding the structural diversity of compounds [6]. CYP450s have been reported to catalyze hydroxylation, isomerization, and coupling reactions in BIAs biosynthesis [9, 10]. CYP450s, involved in the biosynthesis of plant natural products, are commonly located in endoplasmic reticulum along with an *N*-terminal signal peptide, such that the crystallographic structural analysis of CYP450 remains challenging. Only a few studies have been reported on the crystal structures of plant CYP450s [11, 12]. Semi-rational design based on homology modeling and mutation analysis provided an alternative way to investigate the structure-function relationship of enzymes, especially the emergence of Alphafold [13]. Commonly, based on homologous protein sequence alignment and existing knowledge, multiple amino acid residues were rationally selected as targets to construct high-quality mutation libraries [14]. Semi-rational mutation in CYP450s have been engineered to analyze catalytic preference and to improve the catalytic activity involved in the biosynthesis of natural products [15, 16].

The CYP719 family is a member of the CYP71 clan [17], among which CYP719B subfamily and most of the CYP719A subfamily members are involved in the BIAs biosynthesis [18]. For example, CYP719B1 have been reported to catalyze the formation of a phenol-couple involved in salutaridine biosynthesis [19], while CYP719A subfamily members led to methylenedioxy bridge-formation by catalyzing the oxidative cyclization of an ortho-hydroxymethoxy-substituted aromatic ring [20–23]. CYP719As continuously catalyzed the formation of (*S*)-stylopine from (*S*)-scoulerine via (*S*)-cheilanthifoline [18]. Although CYP719As shared approximately 60% of the amino acid sequence identity, they all exhibited high substrate regiospecificity and only catalyzed the methylenedioxy bridge-formation on the A or D rings of protoberberine alkaloids [21–23].

**Fig. 1:**
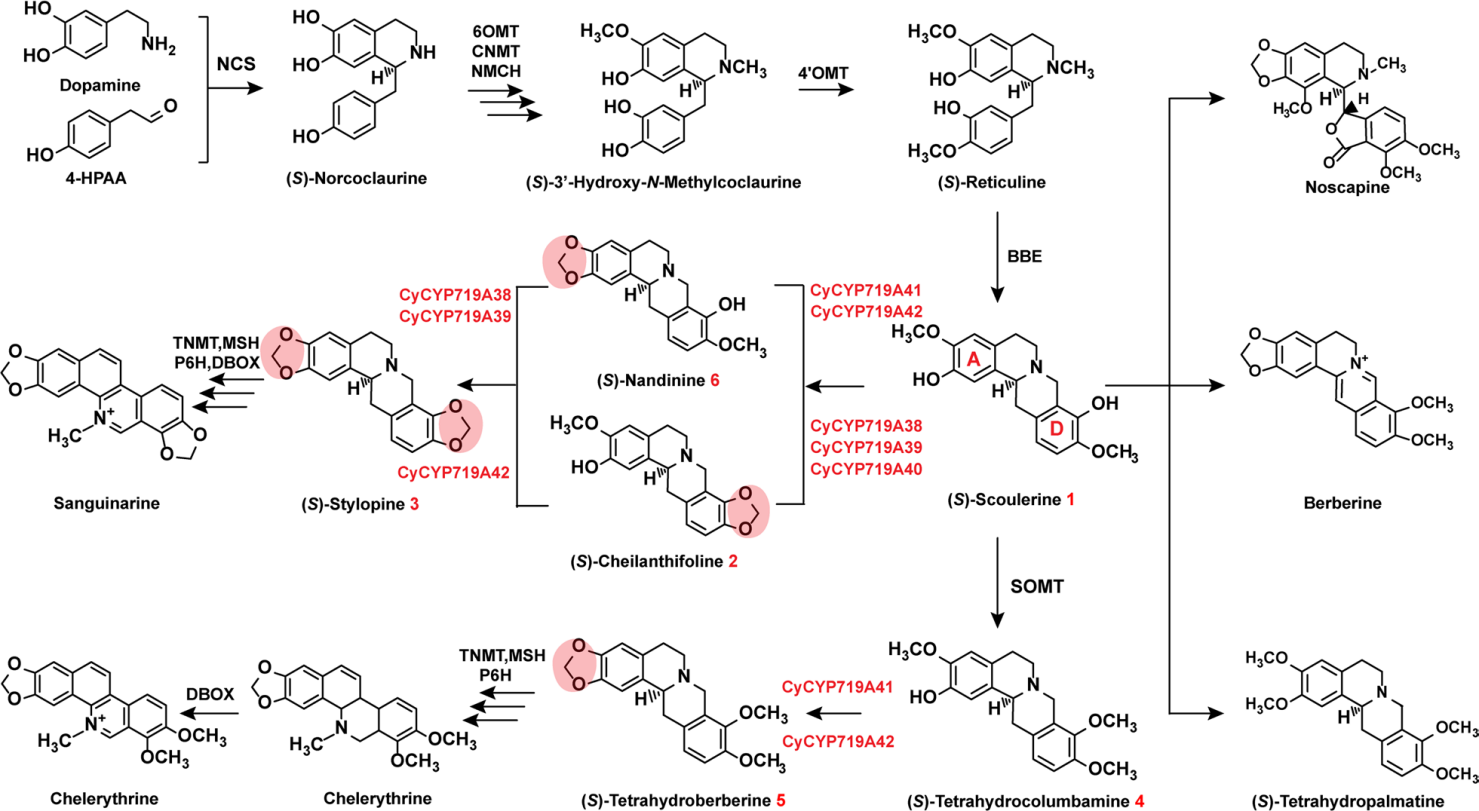
Biosynthetic pathway of benzylisoquinoline alkaloids with methylenedioxy bridge

Currently, CYP719As from Papaveraceae and Ranunculaceae have been identified to catalyze the methylenedioxy bridge-formation on the A or D rings of protoberberine alkaloids [20–24]. However, the key residues related to the substrate regioselectivity is undiscovered. Here, we employed five CyCYP719As from *C. yanhusuo* to explore their regioselectivity. In vitro enzyme assays demonstrated that CyCYP719As functionally catalyzed the methylenedioxy bridge-formation on the A or D rings of protoberberine alkaloids (Fig. 1). To identify the specific residues related to the methylenedioxy bridge-formation on the A and D rings, semi-rational design was employed to engineer the CyCYP719As and identify the major residues associated with regiospecificity. Finally, the functionally characterized CyCYP719As and their mutations were used for de novo production of methylenedioxy bridge compound (*S*)-stylopine in yeast strain by synthetic biology.

## Results

### Identification and phylogenetic analysis of CyCYP719As

Five full-length genes annotated as "CYP719 family" were found in the transcriptome of *C. yanhusuo* [25]. These genes were cloned from *C. yanhusuo* and designated as *CyCYP719A38, CyCYP719A39, CyCYP719A40, CyCYP719A41, CyCYP719A42* (sequences were shown in the Additional file 1: Material S1)*,* following the CYP450 nomenclature committee guidelines. Multiple amino acid sequence alignment revealed the conserved eukaryotic cytochrome P450 region: a helical K region, an aromatic region, and a carbon-terminal heme-binding region [20] (Additional file 1: Figure S1). The conserved amino acid sequence in the I helix of CYP450 was [(A/G) GX (D/E) T (T/S)][26], while in the sequences of CYP719 family, leucine replaced conserved alanine/glycine and serine replaced conserved threonine (Additional file 1: Figure S1) [27, 28].

**Fig. 2:**
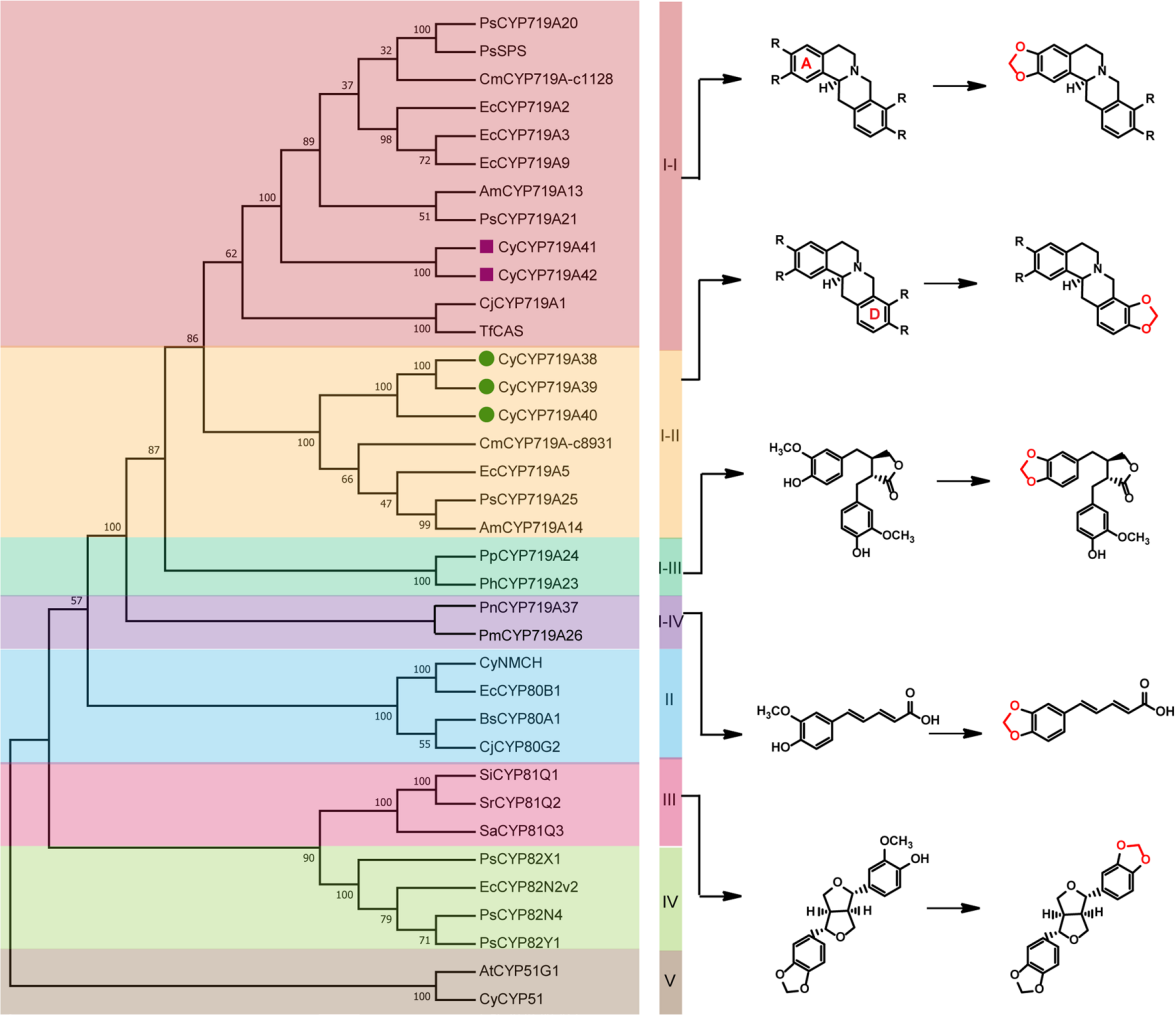
Phylogenetic analysis of functionally characterized CYP450s involved in the methylenedioxy bridge-formation. CyCYP719As from *C. yanhusuo* were indicated by green dots and violet squares. Bootstrap support values for each clade were based on 1000 iterations. GenBank accession numbers were listed in Additional file 1: Table S2

CYP81Qs and CYP719As subfamily genes have been characterized to catalyze the methylenedioxy bridge-formation [21, 29]. Here, phylogenetic analysis was performed for the CYP450s involved in the methylenedioxy bridge-formation (CYP81Qs and CYP719As) and functional genes (CYP80s, CYP82s, and CYP719s) involved in the BIAs biosynthesis. CYP51s from *A. thaliana* and *C. yanhusuo* were selected for the outgroups (Fig. 2). The analysis revealed that CYP719As from different species with approximately 60% identity clustered in a clade, and were divided into four subclades. CYP719As in subclade I-IV were from *Piper* genus, which participated in the methylenedioxy bridge-formation in the biosynthesis of piperic acid and kavalactones, respectively [30, 31]. CYP719As in subclade I-III were from Podophyllum species, which were involved in the methylenedioxy bridge-formation of lignan (-)-pluviatolide [32]. CYP719As, catalyzing the methylenedioxy bridge-formation of protoberberine alkaloids, were clustered together and further divided into two subclades (subclade I-I and I-II). CyCYP719A41 and CyCYP719A42 were clustered with the functional enzymes that catalyzed the methylenedioxy bridge-formation on the A ring of protoberberine alkaloids [20, 23, 33, 34]. CyCYP719A38, CyCYP719A39, and CyCYP719A40 were clustered with the functional genes that catalyzed the methylenedioxy bridge-formation on the D ring of protoberberine alkaloids [21, 22, 35].

### Functional characterization of CyCYP719As

Five CyCYP719As have been functionally characterized to be involved in the methylenedioxy bridge-formation in BIAs biosynthesis. *Saccharomyces cerevisiae* WAT11, engineered to overexpress a CYP450 reductase (CPR) from *A. thaliana*, was utilized to heterogeneously express CyCYP719As [36]. The expression of CyCYP719As was induced by galactose, from which microsomal protein was extracted [37], and different protoberberine alkaloids were selected as substrates to verify the enzyme activity in vitro.

**Fig. 3:**
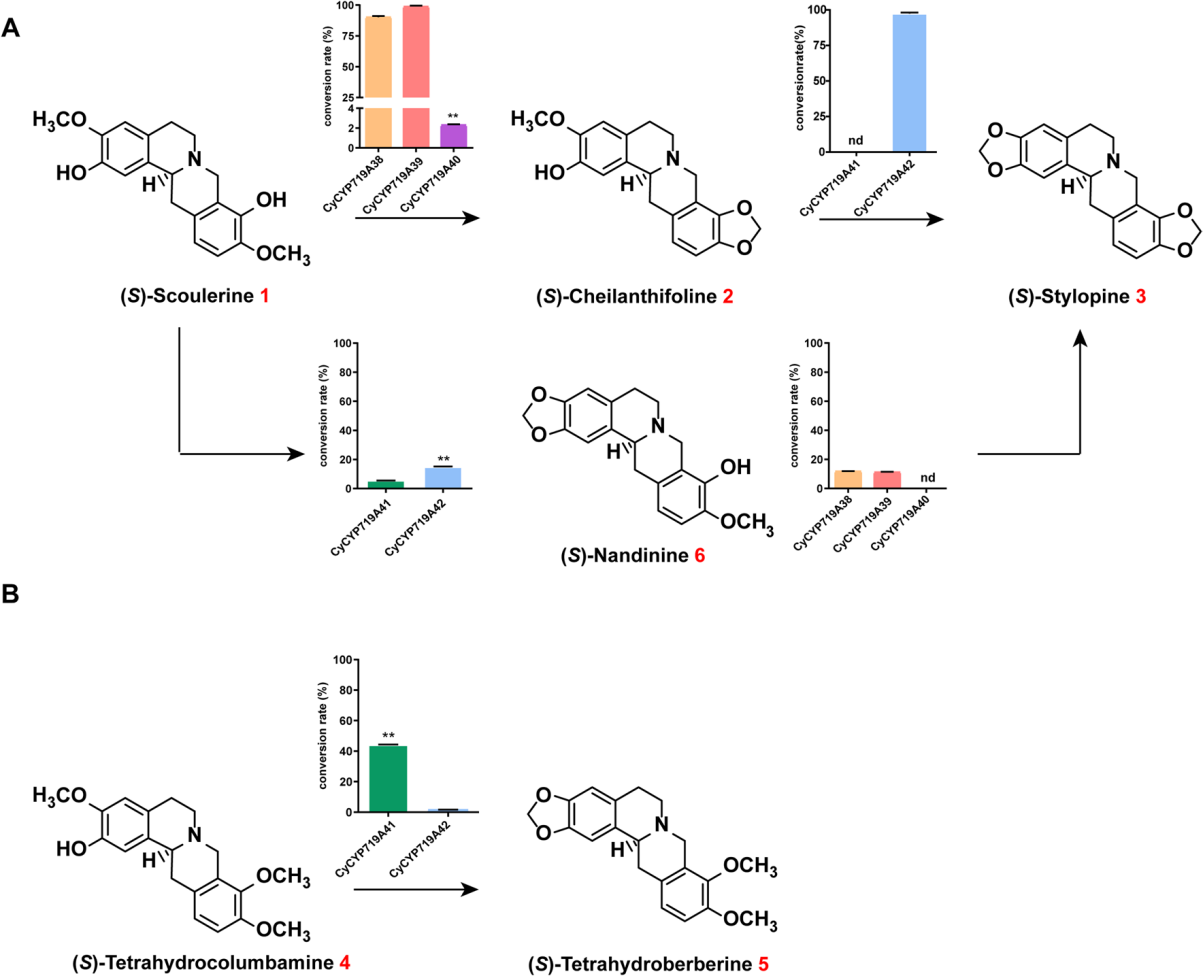
Results of in vitro enzyme assay of CyCYP719As. The column graph indicated the conversion rate relative to the added substrate. **A** CyCYP719As catalyzed the conversion of (*S*)-scoulerine **1** to (*S*)-stylopine **3**. **B** CyCYP719As catalyzed the conversion of (*S*)-tetrahydrocolumbamine **4** to (*S*)-tetrahydroberberine **5**. Data reported are the means ± SD from triplicate analyses. ** indicates P < 0.01; nd, not detected

UPLC-QTOF-MS demonstrated that CyCYP719A38, CyCYP719A39, and CyCYP719A40 could catalyze the methylenedioxy bridge-formation on the D ring of protoberberine alkaloids. They could catalyze the conversion of substrate (*S*)-scoulerine **1** to (*S*)-cheilanthifoline **2** (Additional file 1: Figures S2, S3). In addition, CyCYP719A38 and CyCYP719A39 with 97% identity could both catalyze the formation of (*S*)-nandinine **6** (a compound containing an A ring methylenedioxy bridge structure) to (*S*)-stylopine **3**, while CyCYP719A40 could not (Additional file 1: Figure S2, S3). In vitro conversion rates indicated that CyCYP719A38 and CyCYP719A39 had higher catalytic efficiency than CyCYP719A40 (Fig. 3A, Additional file 1: Table S3). CyCYP719A38 and CyCYP719A39 had approximately the same conversion rate of over 90% when using (*S*)-scoulerine as substrate, while CyCYP719A40 only had a 2% conversion rate (Fig. 3A, Additional file 1: Table S3).

In vitro enzyme activity analysis revealed that CyCYP719A41 and CyCYP719A42 could catalyze the methylenedioxy bridge-formation on the A ring of protoberberine with substrate regioselectivity. The main catalytic activity of CyCYP719A42 was to catalyze the formation of (*S*)-cheilanthifoline **2** to (*S*)-stylopine **3**, with a conversion rate of more than 90% (Fig. 3A, Additional file 1: Figures S2, S3, Table S3). In addition, CyCYP719A41 and CyCYP719A42 could also catalyze (*S*)-scoulerine **1** as a substrate to produce (*S*)-nandinine **6** (Additional file 1: Figures S2, S3). In vitro conversion rates showed that CyCYP719A42 had a relatively higher conversion rate of 15%, which was more efficient than CyCYP719A41 with a conversion rate of 5% (Fig. 3A, Additional file 1: Table S3).

Here, CyCYP719A38, CyCYP719A39, and CyCYP719A40 were characterized as catalyzing methylenedioxy bridge-formation on the D ring of protoberberine, while CyCYP719A41 and CyCYP719A42 could catalyze the methylenedioxy bridge-formation on the A ring. Based on a comparison analysis of the conversion efficiency, it was found that during the catalytic formation of (*S*)-stylopine, the methylenedioxy bridge-formation on the D ring of protoberberine was prior to the methylenedioxy bridge-formation on the A ring. In addition, to determine the substrate regiospecificity of CyCYP719As, we screened 1-benzylisoquinoline and protoberberine alkaloids, including (*S*)-tetrahydrocolumbamine, (*S*)-coclaurine, (*S*)-norcoclaurine, (*S*)-*N*-methylcoclaurine, (*S*)-reticuline, (*S*)-3’-hydroxy-*N*-methylcoclaurine, jatrorrhizine, corytuberine, tetrahydropalmatine, corydaline, and columbamine as substrates (Additional file 1: Figure S4) to characterize the function of CyCYP719As. Except for (*S*)-tetrahydrocolumbamine, none of the compounds could be employed as substrates for CyCYP719As. CyCYP719A41 could efficiently catalyze the formation of (*S*)-tetrahydrocolumbamine **4** to (*S*)-tetrahydroberberine **5**, with a conversion rate of more than 40% (Fig. 3B, Additional file 1: Figure S3, Table S3). Here, CyCYP719As genes with different substrate preferences and conversion rates provided a variety of genetic elements for the BIAs biosynthesis.

### Semi-rational mutation analysis to reveal key residues related to regioselectivity of CyCYP719As

#### Candidate residues selection

In vitro enzyme assays and research reports revealed that the formation from (*S*)-scoulerine to (*S*)-stylopine required the participation of two CYP719As with strict substrate regioselectivity, which catalyzed the methylenedioxy bridge-formation on the D and A rings of protoberberine alkaloids successively [20, 23]. We then sought to find the key residues that catalyzed the reaction of the A and D rings to form the methylenedioxy bridges and affected the catalytic activity. CyCYP719A39 and CyCYP719A42 were selected for mutation analysis due to their relatively high catalytic activity.

**Fig. 4:**
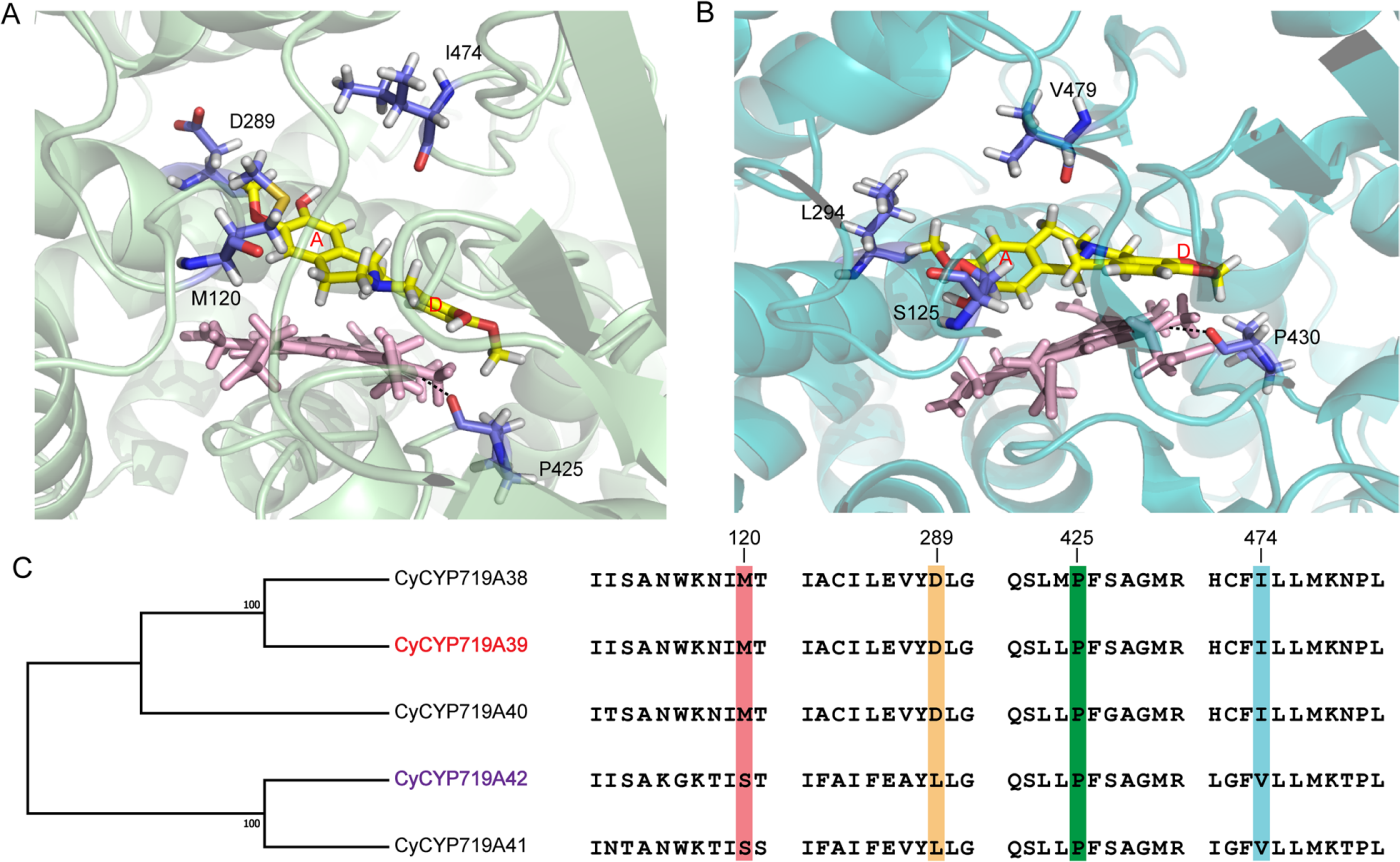
Homology modeling and docking analysis of CyCYP719A39 and CyCYP719A42. **A** Docking view of CyCYP719A39 with (*S*)-scoulerine. **B** Docking view of CyCYP719A42 with (*S*)-cheilanthifoline. Heme was shown as a stick structure with pink. Substrate was shown as a stick structure with yellow. **C** The four selected residues for mutation analysis

Homology modeling and docking analysis of CyCYP719A39 and CyCYP719A42 were employed to search for key residues affecting catalytic activity (Fig. 4A, B). CYP17A1 from humans (PDB ID: 4nkx.4.A) and CYP17A1 from zebrafish (PDB ID: 6b82.1.B) were selected as suitable replacements for the CyCYP719A39 and CyCYP719A42 templates, given their relatively high sequence similarity (26.39% for CYP17A1 and CyCYP719A39, and 24.01% for CYP17A1 and CyCYP719A42). By combining molecular docking results of CyCYP719As and sequence alignment analysis of CYP719As that catalyzed the formation of methylene dioxygen bridge, three residues (Met120, Asp289, and Ile474 in CyCYP719A39, and Ser125, Leu294, and Val479 in CyCYP719A42) were found to be located in the binding site or active center of CyCYP719As that differed between the two CyCYP719As, and were selected as candidates for semi-rational design and analysis (Fig. 4C). In addition, amino acid Pro425, located close to the active site in CyCYP719As, was also selected because of its interaction with the active center heme (Fig. 4A, B). Proline was mutated to alanine with the shortest side chain to observe the functional changes of CyCYP719As. Therefore, four amino acid residues were selected as the candidate active residues for semi-rational design, and a mutant library was constructed for in vitro functional identification.

#### Functional characterization of mutants

**Fig. 5:**
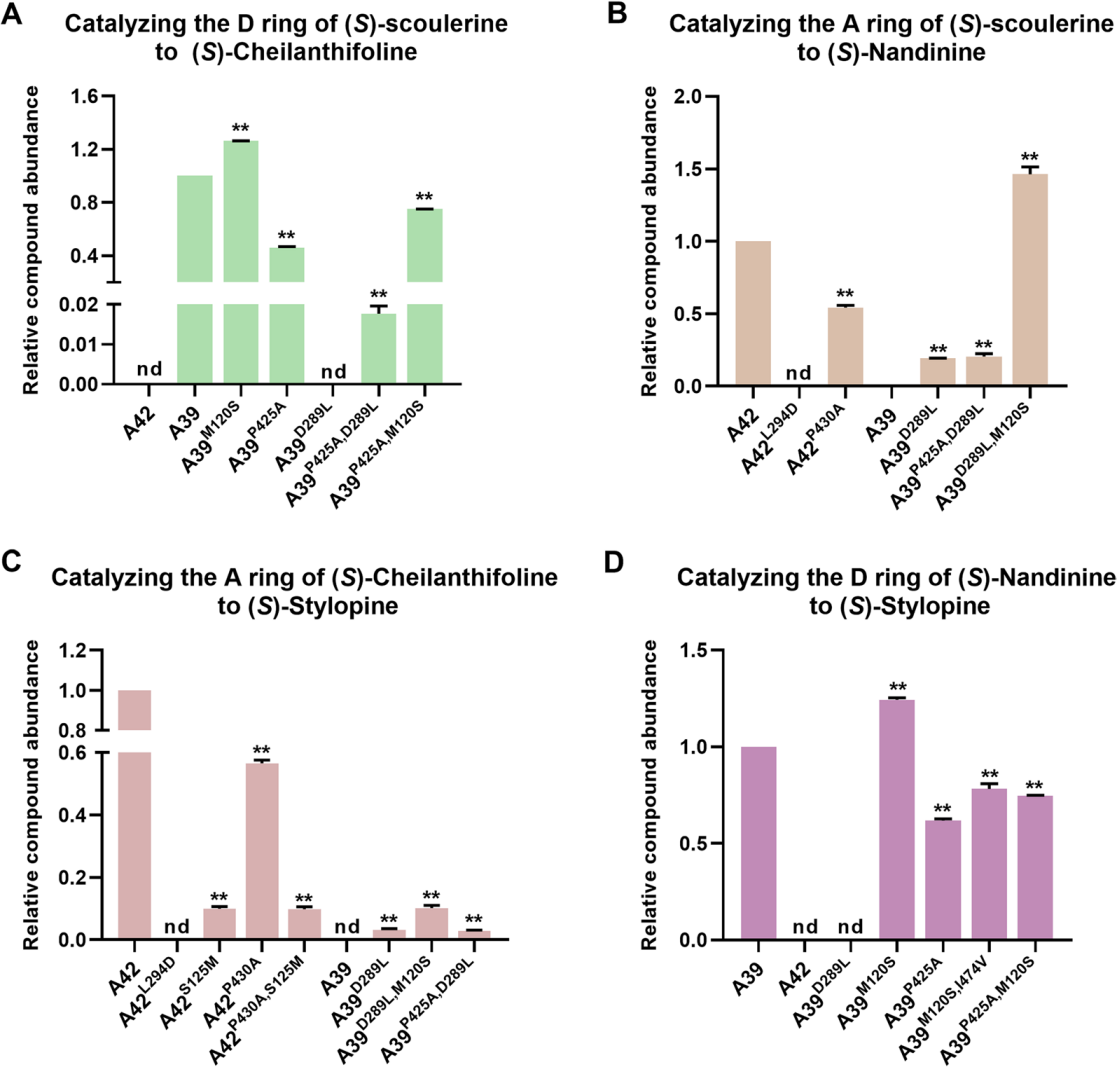
Conversion rates of CyCYP719As mutants relative to wild-type CyCYP719As. **A** Conversion rates of CyCYP719As mutants in catalyzing the D ring formation using (*S*)-scoulerine as substrate to produce (*S*)-cheilanthifoline. **B** Conversion rates of CyCYP719As mutants in catalyzing the A ring formation using (*S*)-scoulerine as substrate to produce (*S*)-nandinine. **C** Conversion rates of CyCYP719As mutants in catalyzing the A ring formation using (*S*)-cheilanthifoline as substrate to produce (*S*)-stylopine. **D** Conversion rates of CyCYP719As mutants in catalyzing the D ring formation using (*S*)-nandinine as substrate to produce (*S*)-stylopine. Data reported are the means ± SD from triplicate analyses. ** indicates P < 0.01; nd, not detected. The experimental data are shown in Additional file 1: Table S4

(*S*)-scoulerine, (*S*)-cheilanthifoline, and (*S*)-nandinine were selected as substrates for in vitro enzyme assays of CyCYP719As. The catalytic activity of CyCYP719As mutant was compared with that of wild-type CyCYP719As (Fig. 5A–D, Additional file 1: Table S4, Figure S5).

**Fig. 6:**
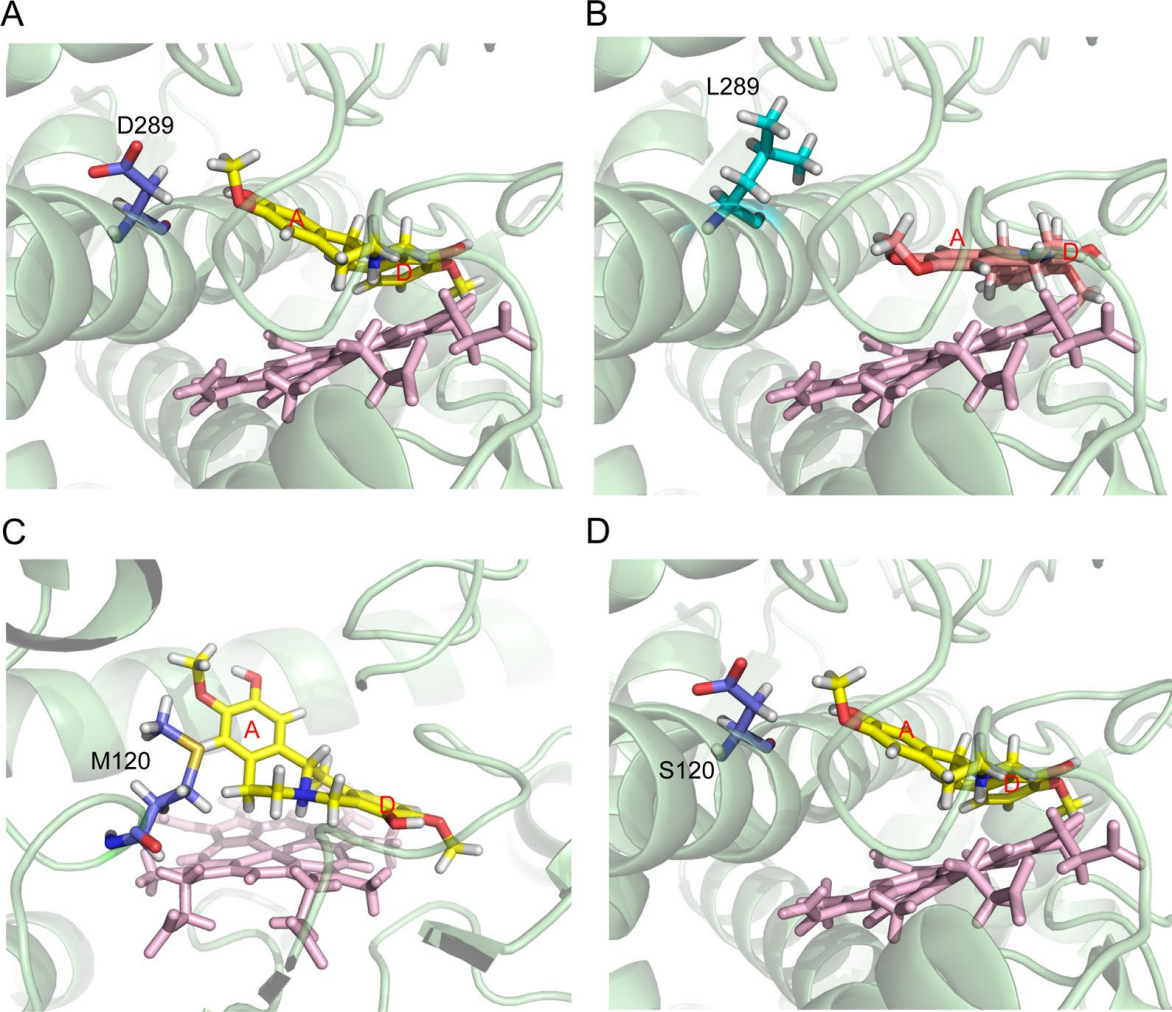
Homology modeling and docking analysis of CyCYP719A39 and its mutants. **A** Docking view of CyCYP719A39 with (*S*)-scoulerine. **B** Docking view of CyCYP719A39^D289L^ mutant with (*S*)-scoulerine. **C** Docking view of CyCYP719A39 with (*S*)-scoulerine. **D** Docking view of CyCYP719A39^M120S^ mutant with (*S*)-scoulerine

The four candidate residues were semi-rationally mutated in CyCYP719A39 and CyCYP719A42. Functional characterization revealed that L294 was essential for CyCYP719A42 to catalyze the methylenedioxy bridge-formation on the A ring of protoberberine, while D289 of CyCYP719A39 was crucial for its ability to catalyze the methylenedioxy bridge-formation on the D ring of protoberberine. L294D mutant of CyCYP719A42 (CyCYP719A42^L294D^) resulted in an inability to catalyze the methylenedioxy bridge-formation on the A ring (Fig. 5C). D289L mutant of CyCYP719A39 (CyCYP719A39^D289L^) catalyzed the methylenedioxy bridge-formation on the A ring, while lost its ability to catalyze the D ring (Fig. 5A, C). We speculated that the hydrophobicity of the active pocket of CyCYP719A39^D289L^ mutant would increase, thereby reducing its mutual exclusion with the substrate A ring, and promoting the reaction between hydroxyl and oxymethyl on the substrate A ring, as well as closing the distance with the heme, which would be more conducive to methylenedioxy bridge-formation on the A ring of protoberberine alkaloids (Fig. 6A–B).

Mutation analysis of CyCYP719A39 indicated that the S120 residue was essential for enhancing the activity of CyCYP719A39 mutants. Introducing the mutation M120S into CyCYP719A39^D289L^ (CyCYP719A39^D289L, M120S^) increased the catalytic conversion rate of methylenedioxy bridge-formation on the A ring more than two times (Fig. 5B, C, Additional file 1: Table S4). M120S mutant of CyCYP719A39 (CyCYP719A39^M120S^) increased the conversion rate of methylenedioxy bridge-formation on the D ring by 20% (Fig. 5A, D, Additional file 1: Table S4). In addition, the S125M mutant of CyCYP719A42 (CyCYP719A42^S125M^) resulted in 90% reduction in catalytic activity for methylenedioxy bridge-formation on the A ring (Fig. 5C, Additional file 1: Table S4). These results were also found to be applicable to the single mutation of S120 residue and combined mutation of multiple residues (Fig. 5A–D, Additional file 1: Figure S5). We speculated that in the CyCYP719A39^M120S^ mutant, the hydrophobicity of the proximal end of the active pocket of the mutant was reduced, which increased the mutual repulsion between the mutant and the substrate A ring, and pulled the substrate D ring closer to the heme, thus promoting the reaction of hydroxyl and oxymethyl on the substrate D ring. However, when mutations D289L and M120S occurred simultaneously, the mutant CyCYP719A39^D289L, M120S^ was more likely to reduce the mutual exclusion with the substrate A ring and to promote a more efficient reaction of the hydroxyl and oxymethyl groups on the substrate A ring.

The methylenedioxy bridge-formation on the D ring is presumed to be prior to the formation on the A ring. We next focused on mutants which could catalyze the formation of methylenedioxy bridges on both the D and A rings of protoberberine alkaloids. Based on the previous analysis, it was indicated that D289 was essential for the methylenedioxy bridge-formation on the D ring of protoberberine catalyzed by CyCYP719A39, and L294 was crucial for methylenedioxy bridge-formation on the A ring catalyzed by CyCYP719A42. Conversion mutations at these residues resulted in the loss of original function for both CyCYP719A39 and CyCYP719A42. However, we found that although the mutant CyCYP719A39^D289L^ completely lost the function of catalyzing the methylenedioxy bridge-formation on the D ring of protoberberine, it did develop the function of catalyzing the methylenedioxy bridge-formation on the A ring. Moreover, double mutant CyCYP719A39^P425A, D289L^ could catalyze the formation of (*S*)-scoulerine to (*S*)-stylopine via (*S*)-cheilanthifoline, although the two reactions could not occur simultaneously (the double mutant cannot directly catalyze the formation of (*S*)-scoulerine to (*S*)-stylopine in vitro*,* requiring stepwise catalysis) (Fig. 5A, C). CyCYP719A39^P425A, D289L^ also catalyzed the formation of (*S*)-scoulerine to (*S*)-nandinine (Fig. 5B), indicating that it could catalyze methylenedioxy bridge-formation on both the A and D rings of (*S*)-scoulerine. Docking analysis showed that P425 in CyCYP719A39 was closest to the active site heme and interacted with it. We speculated that the introduction of P425A mutation reduced the interaction between CyCYP719A39^P425A^ and the substrate, leading to a shift of the D ring away from the heme and thus reducing its catalytic activity. However, the introduction of D289L mutation weakened the mutual exclusion between the double mutant CyCYP719A39^P425A, D289L^ and the substrate A ring, which enabled the double mutant to catalyze the A and D rings of protoberberine alkaloids. Additional iterative mutation analysis based on the double mutant is needed to improve the efficiency of CyCYP719As in catalyzing the methylenedioxy bridge-formation on both the A and D rings of protoberberine.

#### De novo synthesis of (*S*)-stylopine

(*S*)-stylopine has a low content in Papaveraceae such as *C. yanhusuo* and *Chelidonium majus*, while having positive anti-inflammatory and anti-fibrosis physiological activities [4, 5, 38]. However, the presence of two methylenedioxy bridges in (*S*)-stylopine makes chemical synthesis difficult. Therefore, we employed the synthetic biology method to construct an engineered yeast strain to achieve microbiological synthesis of (*S*)-stylopine. In previous work, we constructed a *S. cerevisiae* strain XJ0695, in which the (*S*)-scoulerine biosynthetic pathway was reconstructed and optimized by advanced metabolic engineering and synthetic biology strategies (Data unpublished). The construction of this strain started from the biosynthesis of the first BIAs molecule (*S*)-norcoclaurine by condensation of tyrosine-derived dopamine and 4-HPAA under norcoclaurine synthase (NCS). To improve the production of (S)-norcoclaurine, various optimization attempts have been conducted, including screening the optimal candidates (CYP76AD5 from sugar beet *Beta vulgaris* and CjNCS from *Coptis japonica*), enlarging the tyrosine pool (overexpression of yeast native *ARO1*, *ARO2*, *ARO3*, and expression of EcAROL from *E. coli*, MtPDH1 from *Medicago truncatula*, and two mutants ARO4* (K229L) and ARO7* (G141S)), impairing the undesirable 4-HPAA scattering (deleting *ARI1*, *ADH6*, *YPR1*, *GRE2* and *HFD1*), increasing extra copies of rate-limiting enzymes (3 copies of CjNCS), introducing plant-derived tyrosine pathway, 4-HPAA biosynthesis pathway. Sequentially, we extended the biosynthetic pathway to produce the key intermediate (*S*)-scoulerine.

**Fig. 7:**
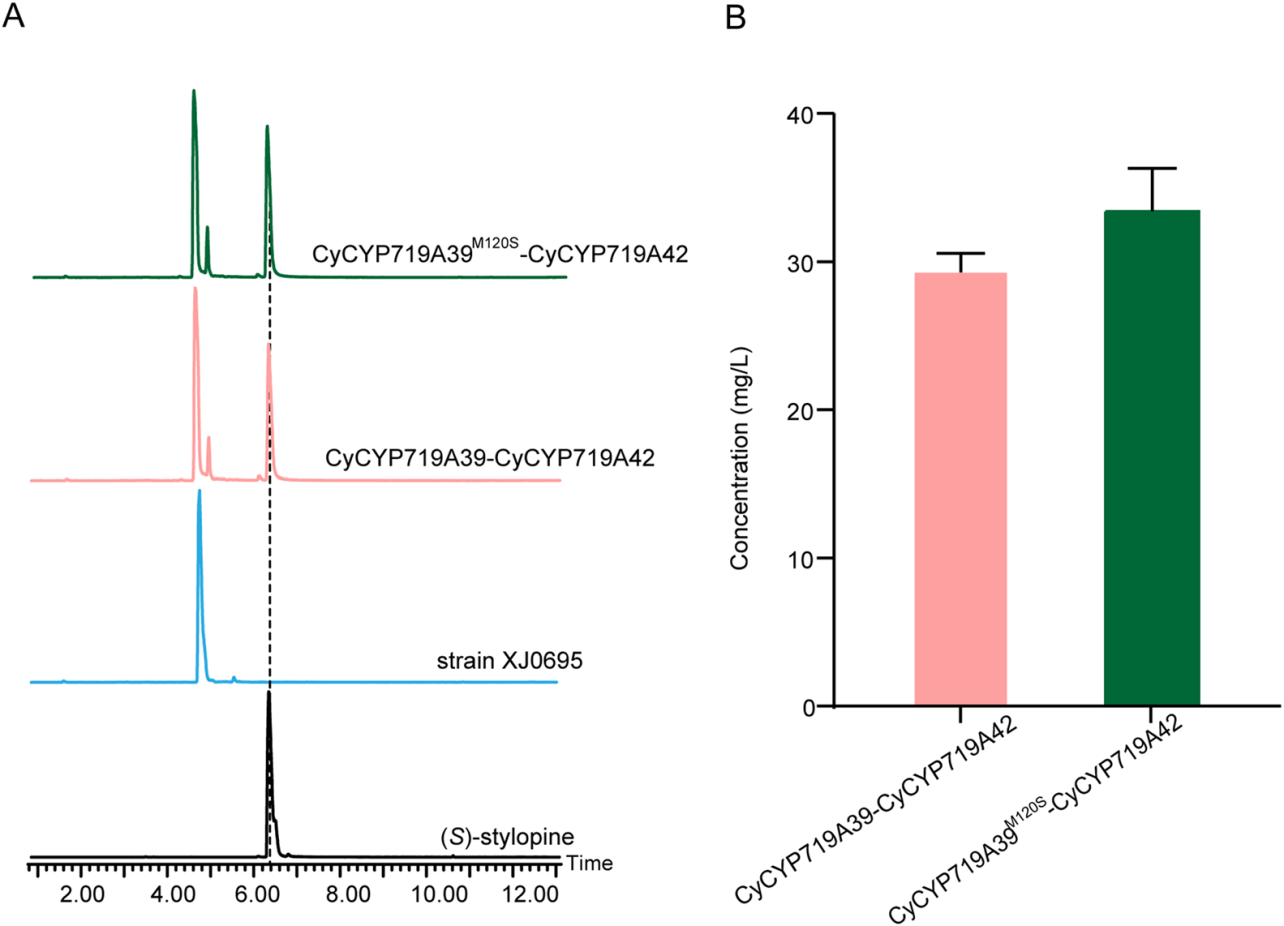
UPLC-MS analysis of (*S*)-stylopine produced by engineered *S. cerevisiae* strains. **A** The extracted ion chromatograms (EICs) of the (*S*)-stylopine produced in yeast strain XJ0695 expression CyCYP719A39-CyCYP719A42 (pink) or CyCYP719A39^M120S^-CyCYP719A42 (green). **B** The production of (*S*)-stylopine in yeast strain XJ0695 expression CyCYP719A39-CyCYP719A42 (pink) or CyCYP719A39^M120S^-CyCYP719A42 (green). Data reported are the means ± SD from triplicate analyses

To construct the yeast strain for de novo synthesis of (*S*)-stylopine, functionally characterized CyCYP719As and mutants were introduced to (*S*)-scoulerine producing yeast platform to synthesize (*S*)-stylopine. For comparison, both CyCYP719A39 and CyCYP719A39^MS^ mutants were paired with CyCYP719A42 to construct recombinant plasmids, which were then introduced into the (*S*)-scoulerine yeast platform containing a copy of *ATR1* from *A. thaliana*. It was induced with 20 g/L galactose after 72 h fermentation in shaker with 20 g/L glucose as carbon source. The results showed that the strain XJ0695 co-expression CyCYP719A39 and CyCYP719A42 could produce (*S*)-stylopine in vivo (Fig. 7A), though accumulated a certain amount of (*S*)-scoulerine. The production of (*S*)-stylopine by the mutant strain CyCYP719A39^MS^-CyCYP719A42 increased approximately 10% to 32 mg/L (Fig. 7B, Additional file 1: Table S5). ​We constructed an engineered yeast strain de novo for (*S*)-stylopine production and further validated the ability of the CyCYP719As mutant to increase the yield of the target product. The method provides a yeast strain for production of plant natural products by synthetic biology. Additional optimization strategies will be executed to improve the conversion of (*S*)-scoulerine to (*S*)-stylopine for high production of those compounds with methylenedioxy bridges.

## Discussion

CYP450 is a crucial modification enzyme in plant natural products biosynthesis, providing groups for the methylation and glycosylation of active compounds [8]. CYP719 is a unique CYP450 family, in which most of the members participate in the biosynthesis of BIAs [34]. In this study, we cloned and identified five CyCYP719A genes from *C. yanhusuo*. In vitro enzyme assays showed that the five genes share approximately 60% identity and catalyzed the methylenedioxy bridge-formation in protoberberine alkaloids, while showing different substrate regiospecificity. CyCYP719A38, CyCYP719A39, and CyCYP719A40 catalyzed the methylenedioxy bridge-formation on the D ring of protoberberine alkaloids followed by the formation of methylenedioxy bridge on the A ring catalyzed by CyCYP719A41 and CyCYP719A42. Key residues related to the methylenedioxy bridge-formation on the A or D rings of protoberberine alkaloids were characterized, providing a reference for the structural–functional relationship of catalytic preference of CyCYP719As. Based on the BIAs production strains, CyCYP719A39, CyCYP719A42 and their mutants were introduced into (*S*)-scoulerine producing yeast platform for de novo synthesis of (*S*)-stylopine with a production of 32 mg/L. This yeast strain provided an alternative for producing (*S*)-stylopine.

Although CYP450s shared different amino acid sequence identities, they contain a common tridimensional fold and catalytic center [39]. This structure made it possible to change the limited residues to alter the catalytic activity, which was helpful for protein engineering of the plant CYP450 without a crystal structure. Without a crystal structure, protein engineering using semi-rational design provided a viable approach to understanding the catalytic mechanism and modification of CYP450. Here, by integrating homology modeling, molecular docking, and sequence alignment, the key residues (Leu294 in CyCYP719A42 and Asp289 in CyCYP719A39) were identified affecting the catalytic substrate regiospecificity of CyCYP719As. We hypothesized that the hydrophobicity of the active pocket of mutant CyCYP719A39^D289L^ would increase after mutation to Leu containing multiple methyl groups compared with Asp with carboxyl groups, reducing its repulsive interaction with the substrate and making the methylenedioxy bridge produced on the A ring closer to the heme, while forcing the D ring away from the heme. Furthermore, functional characterization of CyCYP719A39^D289L^ in the methylenedioxy bridge-formation on the A ring instead of the D ring proved this hypothesis. This function was enhanced after the introduction of M120S into CyCYP719A39^D289L^, which additionally reduced steric hindrance between the A ring and heme. While CyCYP719A42^L294D^ lost its ability to catalyze the methylenedioxy bridge-formation on the A ring of protoberberine. In addition, we found that M120S mutation in CyCYP719A39 could improve the catalytic efficiency by reducing the hydrophobicity of proximal active pocket and narrowing the distance between the D ring and heme. We also obtained a double mutant CyCYP719A39^P425A, D289L^ capable of continuous catalytic reactions, although with a relatively low conversion rate. Further iterative mutation analysis for CyCYP719A39 mutants was needed to identify the mechanism of continuous catalysis and to improve the catalytic efficiency to obtain an efficient mutant protein for continuously catalyzing both A and D rings formation. Afterwards, the output of (*S*)-stylopine product was fine-tuned by the introduction of various CyCYP719As and their mutants into the (*S*)-scoulerine-producing yeast.

In summary, we functionally characterized five CyCYP719As participated in the BIAs biosynthesis and identified key residues related to the methylenedioxy bridge-formation on the A ring (Leu294 in CyCYP719A42) and D ring (Asp289 in CyCYP719A39) by site-directed mutations and in vitro enzyme assays. Finally, an engineered yeast strain for production of (*S*)-stylopine was constructed, which provided an alternative way for producing BIAs compounds. These results extend our knowledge of the structure–function relationship for CYP719As-mediated methylenedioxy bridge-formation and lay the foundation for the BIAs production by synthetic biology.

## Materials and methods

### Plant materials and chemicals

The bulb of *C. yanhusuo* was collected from the traditional production area of Pan'an County, Zhejiang Province, China. The samples were immediately frozen in liquid nitrogen and stored at − 80 °C. (*S*)-scoulerine, (*S*)-stylopine, (*S*)-cheilanthifoline, (*S*)-nandinine, (*S*)-tetrahydrocolumbamine, and (*S*)-tetrahydroberberine with purity ≥ 95% were obtained from Shanghai Yuanye Bio-Technology.

### Cloning of *CyCYP719As* genes

Total RNA was extracted using a Total RNA Kit (TIANGEN, China), and purity and integrity of the RNA were measured. Total RNA from the bulb of *C. yanhusuo* was reverse transcribed into cDNA using Superscript™ IV (Invitrogen, USA). ​According to the transcriptome data obtained in the laboratory, five CyCYP719As subfamily genes were screened, and specific primers were designed based on their open reading frames. *Spe I* restriction site was selected to design primers, as shown in Additional file 1: Table S1. PrimeSTAR^®^ HS DNA Polymerase (Takara, Japan) was utilized for amplification, and the target fragment was obtained by purifying the PCR product using the Quick Gel Extraction Kit (TransGen, China). The target fragment was attached to the eukaryotic expression vector pESC-His by seamless stitching [40]. Plasmid was transformed into *E. coli* Trans1 T1 and the positive clone was confirmed through PCR detection and DNA sequencing. Recombinant plasmid pESC-His-*CyCYP719As* was obtained from the strains using the Plasmid Miniprep Kit (TransGen, China).

### Functional characterization of CyCYP719As

Recombinant plasmid pESC-His-CyCYP719As was expressed in *S. cerevisiae* WAT11, which overexpressed CYP450 reductase (CPR) from *A. thaliana* [36], and then grew to single colony formation at 30°C in defective SD-His solid medium. Positive single colony was inoculated into SD-His liquid medium for culture until the OD_600_ value of the bacterial solution reached 2–3. Then 20 g/L galactose was added to induce protein expression. Microsomal protein was extracted using the TESB method, whose detailed operation was referred to *Ma *et al. [41]. In addition, the blank plasmid pESC-His was transferred into WAT11, and microsomal protein was extracted following to the same procedure for negative control. In vitro functional characterization of CyCYP719As was as follows: reaction system was 500 μL, containing 0.5 mg microsomal protein, 1 mM NADPH, 100 μM substrate, as well as the regenerative system containing 4 mM glucose-6-phosphate (G6P), 1 unit glucose-6-phosphate dehydrogenase (G6P-DH), 5 μM flavin adenine dinucleotide (FAD), 5 μM flavin mononucleotide (FMN), and 2 μM dithiothreitol (DTT). The reaction product was oscillated at 30 °C for 3 h, and then extracted with 500μL ethyl acetate several times. The supernatant was collected and dried with nitrogen, and then dissolved in 200μL methanol for the detection of UPLC-QTOF-MS. ​The protein concentration of the CyCYP719As enzyme in the microsomes was estimated using the extinction coefficient method and used for quantitative studies [40]. In vitro enzyme reaction conversion detection, the enzyme conversion rate was roughly estimated by the product peak area/ (substrate peak area + product peak area) × 100%.

### UPLC-QTOF-MS analysis

The catalytic products were tested by ultra-high performance liquid chromatography-quadrupole time-of-flight mass spectrometry (UPLC-QTOF-MS). The products were separated by Waters Atlantis T3 column (2.1 mm × 100 mm, 1.8 μm). Gradient elution of the enzyme-induced products was performed with mobile phase solvent A composed of 0.1% formic acid (v/v) and 99.9% acetic acid (v/v) and solvent B composed of 0.1% formic acid water (v/v) at a flow rate of 0.4 mL/min. Elution procedure was referred to the paper by *Liu *et al. [40]. The volume of each injection was 1 μL. Waters Xevo G2-S QTOF instrument was employed for mass spectrometry in positive ion mode. The full scan range was 50 to 1000 Da, scan time was 0.1 s, and slope collision energy was 40 to 60 V. MassLynx (Waters Technologies) software was employed for data analysis. All in vitro enzyme activity assays in this experiment were repeated three times.

### Homology modeling and semi-rational design

The AlphaFold2 platform was employed for protein homology modeling of functional genes of CyCYP719A39 and CyCYP719A42, and to find the model with the highest similarity. Filter templates from the PDB database combining alternating sequence alignments and secondary structure predictions, and create multiple models based on these templates [42]. Molecular docking of CyCYP719A39 and CyCYP719A42 with substrates (*S*)-scoulerine and (S)-cheilanthifoline was simulated, and the docking results were displayed by PyMOL software. AutoDock4 software was used for docking analysis of protein models to search for key residues [43]. The pESC-His-CyCYP719A39 and pESC-His-CyCYP719A42 plasmids were employed as templates for amplification, and the primers were shown in Table S1. Recombinant plasmids containing the mutated sequences were constructed by seamless cloning and splicing with the eukaryotic expression vector pESC-His, and transformed into WAT11 for expression. Functional characterization of the mutant was consistent with that of wild-type CyCYP719As in vitro. All in vitro enzyme activity assays were repeated three times.

### Yeast culture assays for (*S*)-stylopine production

According to the *S. cerevisiae* strain XJ0695 that could produce (*S*)-scoulerine provided by Chen Yun's research group and a copy of *ATR1* from *A. thaliana* was integrated into *YORWΔ17*CyCYP719A39 and CyCYP719A39^MS^ mutant were introduced into the engineering yeast together with CyCYP719A42 for functional characterization in vivo and (*S*)-stylopine production. The recombinant pESC-Ura-CyCYP719As were constructed due to the lack of Ura tag in engineered yeast, and then cultured in the Ura-deficient liquid medium after transplanting into engineered yeast. Fermentation was performed in shaker with 20 g/L glucose as the sole carbon source until OD_600_ value reached 3, and 20 g/L galactose was added for induction. The reaction product was extracted with an equal volume of 30% acetonitrile, centrifuged and diluted with 15% acetonitrile, and tested by UPLC-QTOF-MS. The engineered yeast with pESC-Ura was utilized as negative control. All in *vitro* enzyme activity assays were repeated three times.

## Supplementary Information

**Additional file 1: S1.** Amino acid sequences of CyCYP719As. **Figure S1.** Multiple sequence alignment of CYP719s. The conserved CYP719 regions, including helix-K, aromatic regions, and heme-binding regions, are highlighted. The conserved amino acid sequence in the I helix of CYP450 is represented by dashed line, leucine and serine are represented by “*”. All sequence information in the figure was shown in Table S2. **Figure S2.** UPLC-QTOF-MS analysis of the catalytic function of CyCYP719As. A: *In vitro* enzyme assays of CyCYP719As using (*S*)-scoulerine 1 as substrate. B: CyCYP719A41 and CyCYP719A42 catalyze (*S*)-tetrahydrocolumbamine 4 to produce (*S*)-tetrahydroberberine 5. C: CyCYP719A38, CyCYP719A39, and CyCYP719A40 catalyze (*S*)-nandinine 6 to produce (*S*)-stylopine 3. D: CyCYP719A41 and CyCYP719A42 catalyze (*S*)-cheilanthifoline 2 to produce (*S*)-stylopine 3. **Figure S3.** A: Mass spectrum of CyCYP719A41 product (2) compared with that of authentic (*S*)-cheilanthifoline. B: Mass spectrum of CyCYP719A42 product (6) compared with that of authentic (*S*)-nandinine. C: Mass spectrum of CyCYP719A41 product (5) compared with that of authentic (*S*)-tetrahydroberberine. D: Mass spectrum of CyCYP719A39 product (3) compared with that of authentic (*S*)-stylopine. **Figure S4.** Structure of the eleven compounds used for *in vitro* enzymatic assay of CyCYP719As. **Figure S5.** Relative yields of different products *in vitro* enzymatic reaction of CyCYP719A39 and CyCYP719A42 and their mutants with (*S*)-scoulerine, (*S*)-cheilanthifoline, and (*S*)-nandinine as substrates. Data reported are the means ±SD from triplicate analyses ** indicates P<0.01; nd, not detected. Data *in vitro* conversion of CyCYP719A mutants are shown in Table S4. **Table S1.** Primers used in this study. **Table S2.** Sequence information used in the phylogenetic analysis in Fig. 2. **Table S3.** *In vitro* conversion rate of CyCYP719A functional genes. **Table S4.** *In vitro* conversion rate of CyCYP719A mutants. Table S5. Concentration of (*S*)-Stylopine produced by fermentation engineered strains.

## Data Availability

All data generated during this study are included in this article, and all material is available upon request.
