# Supplementary material for: Structure-function analysis of CYP719As involved in methylenedioxy bridge-formation in the biosynthesis of benzylisoquinoline alkaloids and its de novo production
Source: Microb Cell Fact. 2023 Feb 3;22:23. doi: 10.1186/s12934-023-02024-2 (PMC9898898; doi:10.1186/s12934-023-02024-2)
Supplement: Supplementary file 1 — Additional file 1: S1. Amino acid sequences of CyCYP719As. Figure S1. Multiple sequence alignment of CYP719s. The conserved CYP719 regions, including helix-K, aromatic regions, and heme-binding regions, are highlighted. The conserved amino acid sequence in the I helix of CYP450 is represented by dashed line, leucine and serine are represented by “*”. All sequence information in the figure was shown in Table S2. Figure S2. UPLC-QTOF-MS analysis of the catalytic function of CyCYP719As. A: In vitro enzyme assays of CyCYP719As using (S)-scoulerine 1 as substrate. B: CyCYP719A41 and CyCYP719A42 catalyze (S)-tetrahydrocolumbamine 4 to produce (S)-tetrahydroberberine 5. C: CyCYP719A38, CyCYP719A39, and CyCYP719A40 catalyze (S)-nandinine 6 to produce (S)-stylopine 3. D: CyCYP719A41 and CyCYP719A42 catalyze (S)-cheilanthifoline 2 to produce (S)-stylopine 3. Figure S3. A: Mass spectrum of CyCYP719A41 product (2) compared with that of authentic (S)-cheilanthifoline. B: Mass spectrum of CyCYP719A42 product (6) compared with that of authentic (S)-nandinine. C: Mass spectrum of CyCYP719A41 product (5) compared with that of authentic (S)-tetrahydroberberine. D: Mass spectrum of CyCYP719A39 product (3) compared with that of authentic (S)-stylopine. Figure S4. Structure of the eleven compounds used for in vitro enzymatic assay of CyCYP719As. Figure S5. Relative yields of different products in vitro enzymatic reaction of CyCYP719A39 and CyCYP719A42 and their mutants with (S)-scoulerine, (S)-cheilanthifoline, and (S)-nandinine as substrates. Data reported are the means ±SD from triplicate analyses ** indicates P<0.01; nd, not detected. Data in vitro conversion of CyCYP719A mutants are shown in Table S4. Table S1. Primers used in this study. Table S2. Sequence information used in the phylogenetic analysis in Fig. 2. Table S3. In vitro conversion rate of CyCYP719A functional genes. Table S4. In vitro conversion rate of CyCYP719A mutants. Table S5. Concentration of (S)-Stylopine produced [file 12934_2023_2024_MOESM1_ESM.docx]

**Supporting Information**

**Structure-function analysis of CYP719As involved in methylenedioxy bridge-formation in the biosynthesis of benzylisoquinoline alkaloids and its de novo production**

Supplementary Material S1. Amino acid sequences of CyCYP719As.

>CyCYP719A38

MEGSLWIVTATIVVVFVIATMFRKSSSISSKTEWPAGPKKLPVIGNLHQLGGDVLHVVFANLAKVYGSVMTVWVGSWKPMIVISDIDRAWEILVNKSNDYSGRDLPEITKIISANWKNIMTADAGPYWTSLRKGLTGHTLSPTNVASQSHLQERDMNNLIIRMNNQAASNNGIIKPLDHLKEEAVRLLSRLIFGQQFGDEHFVEGIHQALDDLVRISGYASLADAFKFCENLPSHKKSISGVHEILSRVRNLVRPYIVPNPPTNTYLHFLHSQKFTEEVIIACILEVYDLGVDSTAATTVWALTFLVREPDVQEKLYQEIKTVIGDRGTVKVEDVSKMTYLQAVMKETMRMKPIAPMAIPHKAVRETTLMGKKIDKNTVVMVNLYAIHHNTKVYPEPYKFRPERFLAVADGKFGNLKAMEQSLMPFSAGMRICAGMELGKLQYGFALASLVNAFKWTCTADGKLPDMSEDHCFILLMKNPLVAQITPRVN

>CyCYP719A39

MEGSFWIVTATIVVVFVIATMFRKSSSISSKTEWPAGPKKLPVIGNLHQLGGDVLHVVFANLAKVYGSVMTVWVGSWKPMIVISDIDRAWEVLVSKSNDYSGRDLPEITKIISANWKNIMTADAGPYWTSLRKGLTGHTLSPTNVASQSHLQERDMNNLVIRMNNQAASNNGIIKPLDHLKEEAVRLLSRLIFGQQFGDEHFVEGIHQALDDLVRISGYASLADAFKFCENLPSHKKSISGVHEILSRVRNLVRPYIVPNPPTNTYLHFLHSQKFTEEVIIACILEVYDLGVDSTAATTVWALTFLVREPEVQEKLYQEIQTVIGDRGMVKVEDVSKMTYLQAVMKETMRMKPIAPMAIPHKAVRETTLMGKKIDKNTVVMVNLYAIHHNPTVYPEPYKFRPERFLAGGDGKFGNLKAIEQSLLPFSAGMRICAGMELGKLQYGFALASLVNAFKWTCTADGKLPDMSEDHCFILLMKNPLVARITPRVN

>CyCYP719A40

MEEIFWVVTATIVVVFVIATTFGKSSSIPSKTEWPAGPIKLPVIGNLHQLGGDVFHVVLANLAKVYGTAMTVWFGSWKPIIIISDIDNAWEVLVNKSNDYAGRDLPEITRITSANWKNIMTADAGPYWSTLRKGLTGHTLAPTNVASQSHLQEKDMNSLISRMRNQAASNNGIIKPLGHLKEETIRLLSRLMFGQHFEDGHFVEGIHQALDDLIRISGYASLADAFKFCENLPSHKKSISEVHNILSRVRNLVRPYIVPNPPTNTYLHFLQSQKFTEEVIIACILEVYDLGIHSTAATTVWALTFLVREPEVQEKLYREIQTVIGDKGTVKVEDVSKMTYLQAVMKETMRMKPIAPMAIPHKVVRETTLMGNKIDKNTVVMVNLFAIHHNPRVYPEPYKFRPERFLADEAGKFGNLKAMEQSLLPFGAGMRICAGMELGKLQYKFALASLVNAFKWSCTVDGKLPDMSEDHCFILLMKNPLVAQITPRLN

>CyCYP719A41

MFVAVVEILKDKAWLLPATLVAVIAIAKIFLGRSSTMKWPTGPKTLPIIGNMHQLGGTDLQVVLAGLAQTYGNIMTIWVGSWRPMIVVSDLQKAWEVLVNKSSDYSGRAMPDITQINTANWKTISSSDSGPHWSNLRKGLQNIALSPHNIAAQSRFQEGDISKMIQTLKQEAVANSGIVHPLDHLKKTTVRLISRLIFGQDFDDDAYVEEMHHVLEELIRISGYARLADAFYYAKYLPSHKQAVDGSWEVNRRVKLMVKPLLSVNPPTNCYLHFLRSQDYSEEVIIFAIFEVYLLGVDSTSSTTAWALGFLIREPRVQEKLYQELKNFAGENGMIKVEDINKLPYLQAVLKETLRMKPIAPLAIPHKAVRETSLVGNKVETGTRVMVNIYAIQHNPKVWIDPYKFKPERFLHGEDNDGGNLKVMEQSLLPFSAGMRTCAGMELGKLQFGFSLANLVNAFQWDCAKKGKLPDMSDLLGFVLLMKTPLEAKIVPRGRGSSPNIN

>CyCYP719A42

MFEAVAEILTEKPWLLPATLVALIAIAKILLGKSSTMKWPTGPKTLPIIGNMHQLGGTDLQVVLANLAKTYGTIMTIWVGSWRPMIVLSDIEKAWEVLVNKSSDYSGRAMPEITEIISAKGKTISTSDSGPHWSNLRKGLQNVALSPHNIAAQSRFQENDITKMIKTLKQEAASNNGIVQPLDHLKKSTVRLISRLIFGQGFDDDEYVEAMHLAVEELIRVSGYARLAEAFYYAKYLPSHKRAVNEVWEANRRVKRIVKPRLSANPPPNCYLHFLSSQDYSEEMIIFAIFEAYLLGVDSTSSTTAWALGFLIREPKVQERLYQELKNCAGESGIIKVEEINKLPYLQAVLKETMRMKPIAPLAIPHKAVRETSLAGNKVEPGTRVMVNLYAIHHNPKVWIDPHKFKPERFLQGEENGGGNLKLMEQSLLPFSAGMRTCAGMELGKLQFGFSLANLVNAFQWDCAKKGIFPDMSDLLGFVLLMKTPLKAKIVPRGSTSINGY


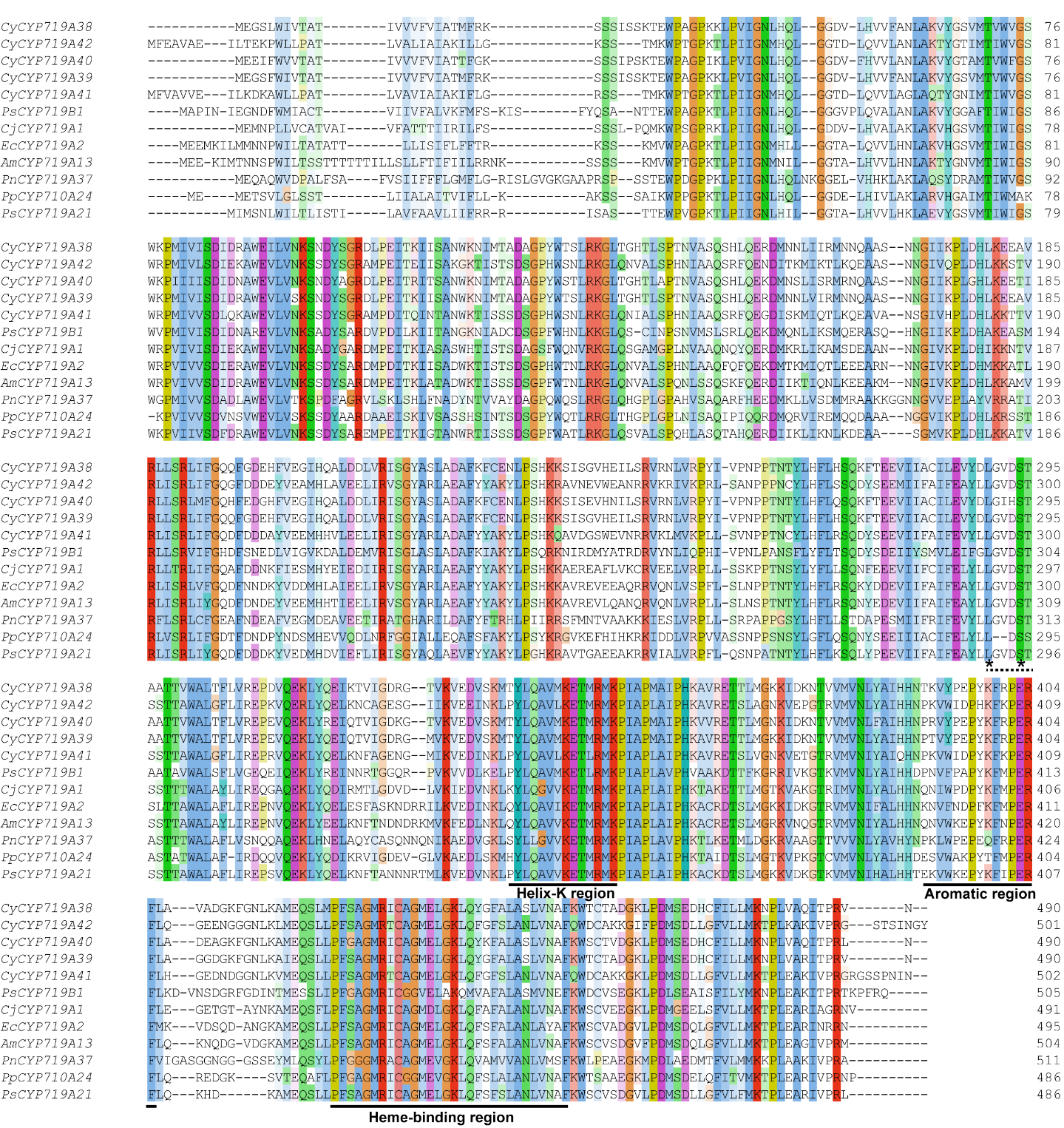


Figure S1. Multiple sequence alignment of CYP719s. The conserved CYP719 regions, including helix-K, aromatic regions, and heme-binding regions, are highlighted. The conserved amino acid sequence in the I helix of CYP450 is represented by dashed line, leucine and serine are represented by “*****”. All sequence information in the figure was shown in Table S2.


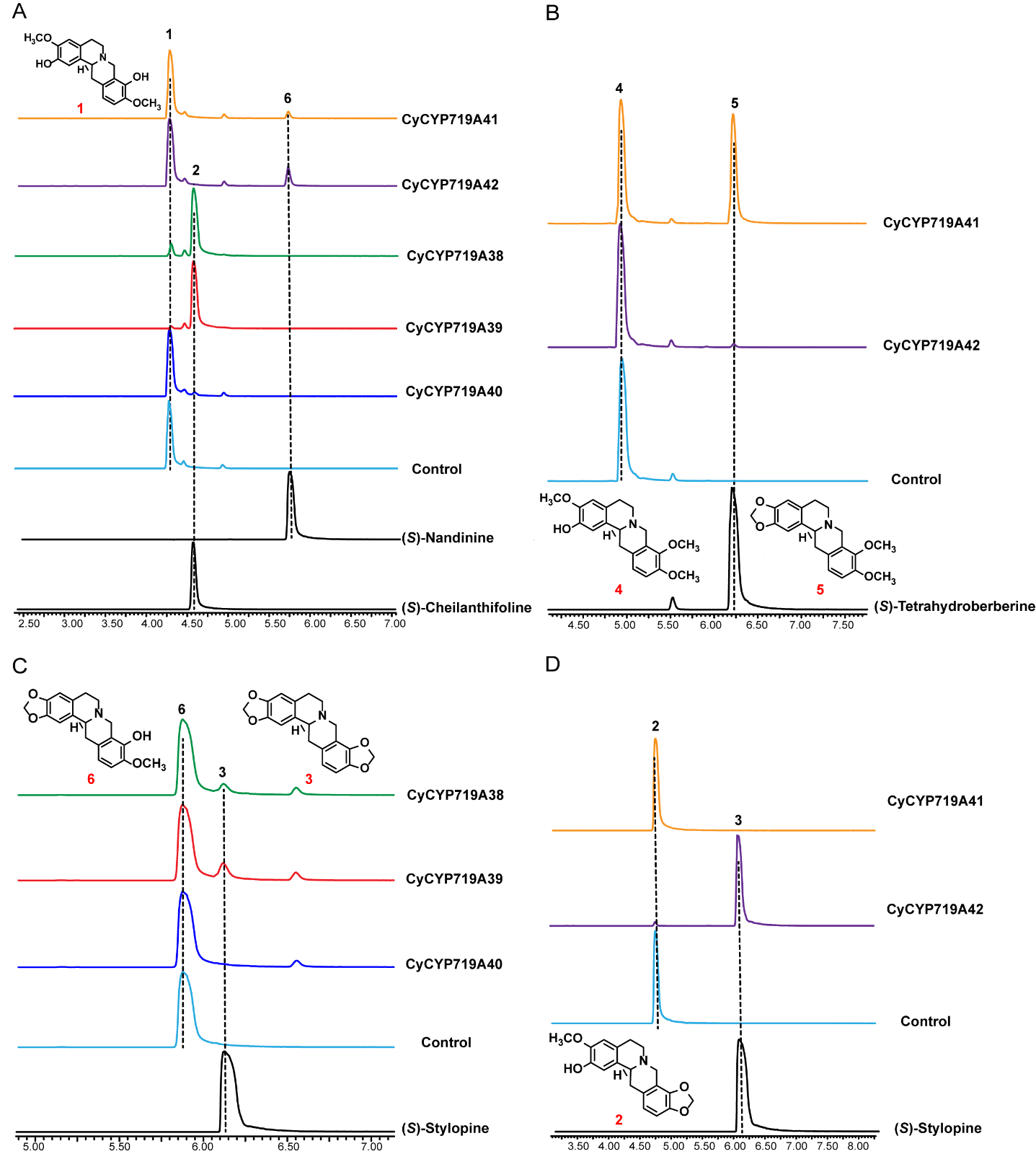


Figure S2. UPLC-QTOF-MS analysis of the catalytic function of CyCYP719As. A: *In vitro* enzyme assays of CyCYP719As using (*S*)-scoulerine **1** as substrate. B: CyCYP719A41 and CyCYP719A42 catalyze (*S*)-tetrahydrocolumbamine **4** to produce (*S*)-tetrahydroberberine **5**. C: CyCYP719A38, CyCYP719A39, and CyCYP719A40 catalyze (*S*)-nandinine **6** to produce (*S*)-stylopine **3**. D: CyCYP719A41 and CyCYP719A42 catalyze (*S*)-cheilanthifoline **2** to produce (*S*)-stylopine **3**.


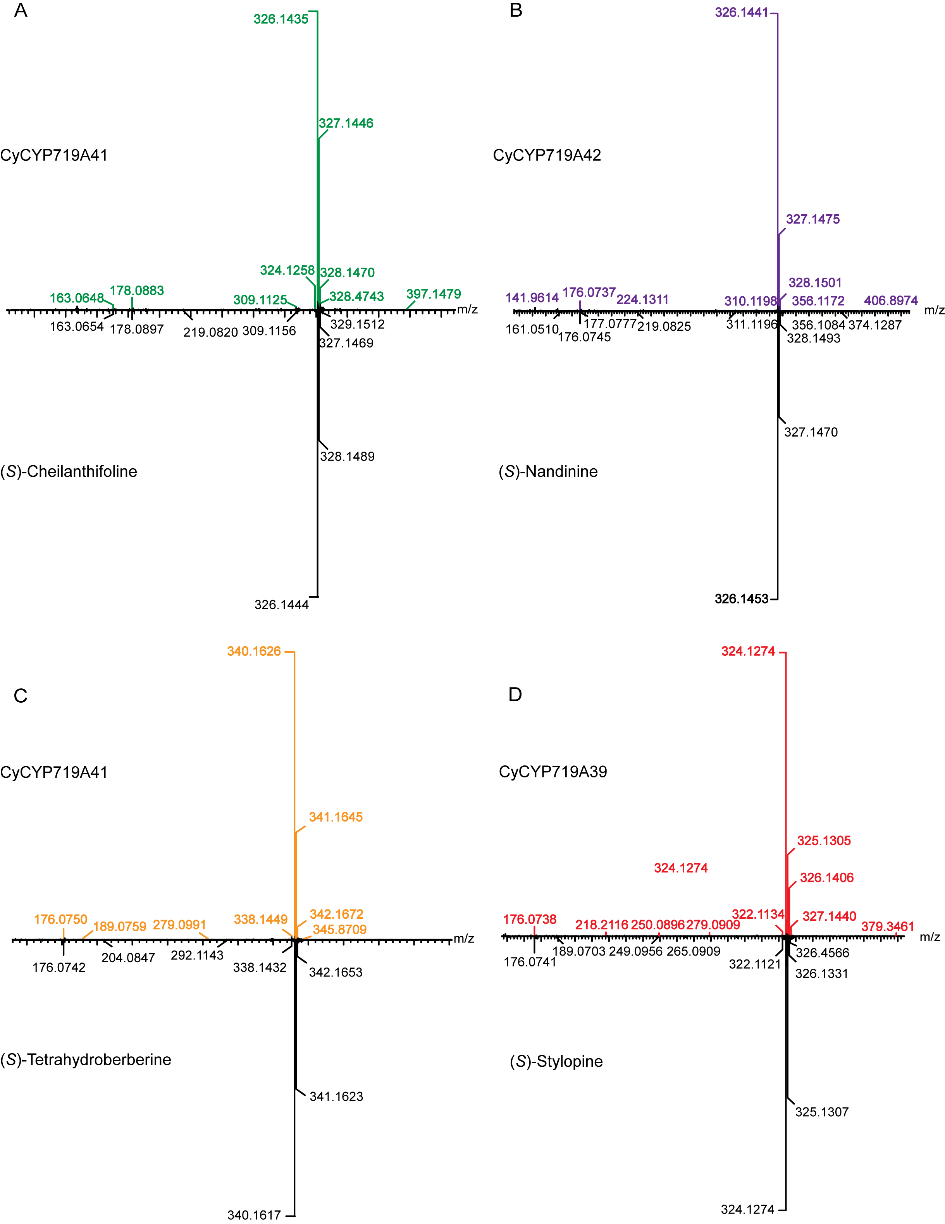


Figure S3. A: Mass spectrum of CyCYP719A41 product (2) compared with that of authentic (*S*)-cheilanthifoline. B: Mass spectrum of CyCYP719A42 product (6) compared with that of authentic (*S*)-nandinine. C: Mass spectrum of CyCYP719A41 product (5) compared with that of authentic (*S*)-tetrahydroberberine. D: Mass spectrum of CyCYP719A39 product (3) compared with that of authentic (*S*)-stylopine.





Figure S4. Structure of the eleven compounds used for *in vitro* enzymatic assay of CyCYP719As.


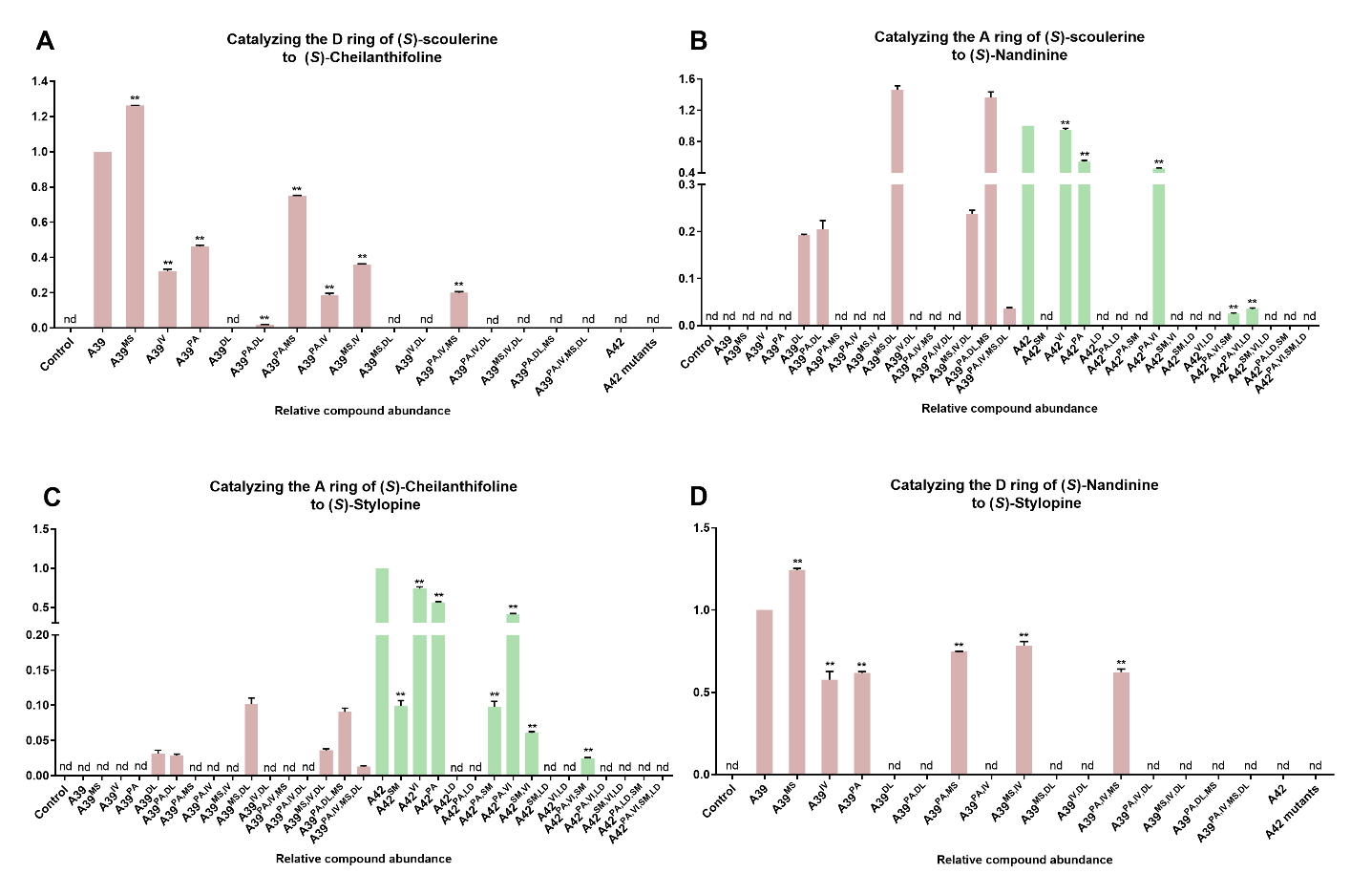


Figure S5. Relative yields of different products *in vitro* enzymatic reaction of CyCYP719A39 and CyCYP719A42 and their mutants with (*S*)-scoulerine, (*S*)-cheilanthifoline, and (*S*)-nandinine as substrates. Data reported are the means ±SD from triplicate analyses ** indicates P<0.01; nd, not detected. Data *in vitro* conversion of CyCYP719A mutants are shown in Table S4.

Table S1. Primers used in this study.

| Name | Sequences (5'-3') |
| --- | --- |
| CyCYP719A38-F | CACTAAAGGGCGGCCGCACTAGTATGGAGGGGAGTCTTTGGATTG |
| CyCYP719A38-R | CTTGTAATCCATCGATACTAGTTTAATTGACTCGAGGAGTAATTTG |
| CyCYP719A42-F | CACTAAAGGGCGGCCGCACTAGTATGTTTGAAGCAGTTGCAGAG |
| CyCYP719A42-R | CTTGTAATCCATCGATACTAGTCTAGTAACCATTAATGGAGGTTG |
| CyCYP719A40-F | CACTAAAGGGCGGCCGCACTAGTATGGAGGAGATTTTTTGGGTTG |
| CyCYP719A40-R | CTTGTAATCCATCGATACTAGTTTAATTGAGTCGGGGAGTAATTTG |
| CyCYP719A39-F | CACTAAAGGGCGGCCGCACTAGTATGGAGGGGAGTTTTTGG |
| CyCYP719A39-R | CTTGTAATCCATCGATACTAGTTTAATTGACTCGAGGAGTAATTC |
| CyCYP719A41-F | CACTAAAGGGCGGCCGCACTAGTATGTTTGTAGCTGTTGTAG |
| CyCYP719A41-R | CTTGTAATCCATCGATACTAGTCTAATTAATATTGGGGGATG |
| CyCYP719A42-L-D-F2 | AAGCATACGACCTCGGTGTTGATAG |
| CyCYP719A42-L-D-R2 | CACCGAGGTCGTATGCTTCGAAG |
| CyCYP719A42-S-M-F2 | AAACGATTATGACTTCTGATTCTGG |
| CyCYP719A42-S-M-R2 | AGAAGTCATAATCGTTTTCCCTTTC |
| CyCYP719A42-V-I-F2 | GGGTTTATACTGTTGATGAAGACACCGCTGAAAGCAAAAATTGTTCCTAGGGGATCAACCTCCATTAATGGTTACTAG |
| CyCYP719A42-V-I-R2 | ATCAACAGTATAAACCCAAGCAAATC |
| CyCYP719A42-P-A-F2 | ACTGTTGGCATTCAGTGCTGGAATG |
| CyCYP719A42-P-A-R2 | CACTGAATGCCAACAGTGATTGCTC |
| CyCYP719A39-D-L-F2 | AGGTTTATCTTCTTGGTGTTGATAG |
| CyCYP719A39-D-L-R2 | ACACCAAGAAGATAAACCTCTAAAATAC |
| CyCYP719A39-M-S-F2 | AGAATATCAGTACAGCAGATGCAGG |
| CyCYP719A39-M-S-R2 | TGCTGTACTGATATTCTTCCAATTTG |
| CyCYP719A39-I-V-F2 | ATTGCTTCGTTTTGTTGATGAAGAACCCACTCGTAGCTCGAATTACTCCTCGAGTCAATTAA |
| CyCYP719A39-I-V-R2 | ATCAACAAAAGGAAGCAATGATCTTC |
| CyCYP719A39-A-P-F2 | ACTTTTGGCATTCAGTGCTGGTATG |
| CyCYP719A39-A-P-R2 | CACTGAATGCCAAAAGTGACTGCTC |

Table S2. Sequence information used in the phylogenetic analysis in Fig. 2.

| Name | *Source* | GenBank accession number |
| --- | --- | --- |
| CjCYP719A1 | *Coptis japonica* | AB026122 |
| EcCYP719A5 | *Eschscholzia californica* | AB434654 |
| EcCYP719A9 | *Eschscholzia californica* | AB434655 |
| AmCYP719A13 | *Argemone mexicana* | EF451151 |
| AmCYP719A14 | *Argemone mexicana* | EF451152 |
| CmCYP719A-c1128 | *Chelidonium majus* | MF039641 |
| CmCYP719A-c8931 | *Chelidonium majus* | MF039640 |
| PsCYP719A25 | *Papaver somniferum* | ADB89213 |
| PsCYP719A20 | *Papaver somniferum* | AHF65153 |
| EcCYP719A2 | *Eschscholzia californica* | AB126257 |
| EcCYP719A3 | *Eschscholzia californica* | AB126256 |
| PnCYP719A37 | *Piper nigrum* | MT643912 |
| PhCYP719A23 | *Podophyllum hexandrum* | KC110997 |
| PpCYP719A24 | *Podophyllum peltatum* | KC110998 |
| PsCYP719A21 | *Papaver somniferum* | JQ659003 |
| TfCAS | *Thalictrum flavum* | AAU20771 |
| PsSPS | *Papaver somniferum* | ADB89214 |
| SiCYP81Q1 | *Sesamum indicum* | AB194714 |
| SrCYP81Q2 | *Sesamum radiatum* | AB194715 |
| SaCYP81Q3 | *Sesamum alatum* | AB194716 |
| AtCYP51G1 | *Arabidopsis thaliana* | NM_101040.4 |
| BsCYP80A1 | *Berberis stolonifera* | P47195 |
| CjCYP80G2 | *Coptis japonica* | AB288053 |
| PsCYP82N4 | *Papaver somniferum* | KC154003 |
| EcCYP82N2v2 | *Eschscholzia californica* | AB598834 |
| PsCYP82X1 | *Papaver somniferum* | I3V6B7 |
| PsCYP82Y1 | *Papaver somniferum* | QBG82621 |
| EcCYP80B1 | *Eschscholzia californica* | AF014801 |
| PsCYP719B1 | *Papaver somniferum* | EF451150 |

Table S3. *In vitro* conversion rate of CyCYP719A functional genes

| Catalytic reaction | Gene | Three replications | | | Conversion rate (%) |
| --- | --- | --- | --- | --- | --- |
| (*S*)-scoulerine to  (*S*)-cheilanthifoline | CyCYP719A38 | 90.3284 | 89.1016 | 91.0480 | 90.16±0.9841 |
|  | CyCYP719A39 | 98.9128 | 98.0269 | 99.5140 | 98.82±0.0663 |
|  | CyCYP719A40 | 2.3768 | 2.2442 | 2.3158 | 2.31±0.7481 |
| (*S*)-scoulerine to  (*S*)-nandinine | CyCYP719A41 | 5.4288 | 5.5544 | 5.2880 | 5.42±0.1333 |
|  | CyCYP719A42 | 14.3784 | 15.2619 | 14.7046 | 14.78±0.4468 |
| (*S*)-cheilanthifoline to (*S*)-stylopine | CyCYP719A42 | 97.9874 | 97.6113 | 96.5432 | 97.38±0.7492 |
| (*S*)-nandinine to  (*S*)-stylopine | CyCYP719A38 | 11.8780 | 11.8792 | 11.7084 | 11.82±0.0983 |
|  | CyCYP719A39 | 11.0550 | 11.2428 | 11.4370 | 11.24±0.1910 |
| (*S*)-tetrahydrocolumbamine to  (*S*)-tetrahydroberberine | CyCYP719A41 | 44.4100 | 43.7871 | 44.0098 | 44.07±0.3156 |
|  | CyCYP719A42 | 1.5162 | 1.5755 | 1.5826 | 1.56±0.0365 |

Table S4. *In vitro* conversion rate of CyCYP719A mutants

| Catalytic reaction | Gene | Relative compound abundance | | | Mean value |
| --- | --- | --- | --- | --- | --- |
| (*S*)-scoulerine to (*S*)-cheilanthifoline | Control | 0.0000 | 0.0000 | 0.0000 | 0.0000 |
|  | CyCYP719A39 | 1.0000 | 1.0000 | 1.0000 | 1.0000 |
|  | CyCYP719A39^DL^ | 0.0000 | 0.0000 | 0.0000 | 0.0000 |
|  | CyCYP719A39^MS^ | 1.2627 | 1.2623 | 1.2630 | 1.2627±0.0004 |
|  | CyCYP719A39^IV^ | 0.3275 | 0.3093 | 0.3302 | 0.3223±0.0113 |
|  | CyCYP719A39^PA^ | 0.4526 | 0.4645 | 0.4667 | 0.4613±0.0076 |
|  | CyCYP719A39^PA,DL^ | 0.0153 | 0.0186 | 0.0189 | 0.0176±0.0020 |
|  | CyCYP719A39^PA,MS^ | 0.7519 | 0.7511 | 0.7504 | 0.7511±0.0008 |
|  | CyCYP719A39^PA.IV^ | 0.1981 | 0.1784 | 0.1816 | 0.1860±0.0107 |
|  | CyCYP719A39^MS,IV^ | 0.3636 | 0.3545 | 0.3596 | 0.3592±0.0046 |
|  | CyCYP719A39^MS,DL^ | 0.0000 | 0.0000 | 0.0000 | 0.0000 |
|  | CyCYP719A39^IV,DL^ | 0.0000 | 0.0000 | 0.0000 | 0.0000 |
|  | CyCYP719A39^PA,IV,MS^ | 0.1924 | 0.2009 | 0.2045 | 0.1993±0.006 |
|  | CyCYP719A39^PA,IV,DL^ | 0.0000 | 0.0000 | 0.0000 | 0.0000 |
|  | CyCYP719A39^MS,IV,DL^ | 0.0000 | 0.0000 | 0.0000 | 0.0000 |
|  | CyCYP719A39^PA,DL,MS^ | 0.0000 | 0.0000 | 0.0000 | 0.0000 |
|  | CyCYP719A39^PA,IV,MS,DL^ | 0.0000 | 0.0000 | 0.0000 | 0.0000 |
|  | CyCYP719A42 | 0.0000 | 0.0000 | 0.0000 | 0.0000 |
|  | CyCYP719A42 mutants | 0.0000 | 0.0000 | 0.0000 | 0.0000 |
| (S)-scoulerine to (S)-nandinine | Control | 0.0000 | 0.0000 | 0.0000 | 0.0000 |
|  | CyCYP719A39 | 0.0000 | 0.0000 | 0.0000 | 0.0000 |
|  | CyCYP719A39^MS^ | 0.0000 | 0.0000 | 0.0000 | 0.0000 |
|  | CyCYP719A39^IV^ | 0.0000 | 0.0000 | 0.0000 | 0.0000 |
|  | CyCYP719A39^PA^ | 0.0000 | 0.0000 | 0.0000 | 0.0000 |
|  | CyCYP719A39^DL^ | 0.1911 | 0.1902 | 0.1946 | 0.1920±0.0023 |
|  | CyCYP719A39^PA,DL^ | 0.1840 | 0.2134 | 0.2183 | 0.2052±0.0186 |
|  | CyCYP719A39^PA,MS^ | 0.0000 | 0.0000 | 0.0000 | 0.0000 |
|  | CyCYP719A39^PA,IV^ | 0.0000 | 0.0000 | 0.0000 | 0.0000 |
|  | CyCYP719A39^MS,IV^ | 0.0000 | 0.0000 | 0.0000 | 0.0000 |
|  | CyCYP719A39^MS,DL^ | 1.4113 | 1.4638 | 1.5120 | 1.4624±0.0504 |
|  | CyCYP719A39^IV,DL^ | 0.0000 | 0.0000 | 0.0000 | 0.0000 |
|  | CyCYP719A39^PA,IV,MS^ | 0.0000 | 0.0000 | 0.0000 | 0.0000 |
|  | CyCYP719A39^PA,IV,DL^ | 0.0000 | 0.0000 | 0.0000 | 0.0000 |
|  | CyCYP719A39^MS,IV,DL^ | 0.2293 | 0.2425 | 0.2422 | 0.2380±0.0075 |
|  | CyCYP719A39^PA,DL,MS^ | 1.4395 | 1.3529 | 1.3098 | 1.3674±0.0660 |
|  | CyCYP719A39^PA,IV,MS,DL^ | 0.0378 | 0.0365 | 0.0348 | 0.0364±0.0015 |
|  | CyCYP719A42 | 1.0000 | 1.0000 | 1.0000 | 1.0000 |
|  | CyCYP719A42^SM^ | 0.0000 | 0.0000 | 0.0000 | 0.0000 |
|  | CyCYP719A42^VI^ | 0.9367 | 0.9418 | 0.9714 | 0.9500±0.0187 |
|  | CyCYP719A42^PA^ | 0.5547 | 0.5468 | 0.5259 | 0.5425±0.0149 |
|  | CyCYP719A42^LD^ | 0.0000 | 0.0000 | 0.0000 | 0.0000 |
|  | CyCYP719A42^PA,LD^ | 0.0000 | 0.0000 | 0.0000 | 0.0000 |
|  | CyCYP719A42^PA,SM^ | 0.0000 | 0.0000 | 0.0000 | 0.0000 |
|  | CyCYP719A42^PA,VI^ | 0.4639 | 0.4530 | 0.4486 | 0.4552±0.0079 |
|  | CyCYP719A42^SM,VI^ | 0.0000 | 0.0000 | 0.0000 | 0.0000 |
|  | CyCYP719A42^SM,LD^ | 0.0000 | 0.0000 | 0.0000 | 0.0000 |
|  | CyCYP719A42^VI,LD^ | 0.0000 | 0.0000 | 0.0000 | 0.0000 |
|  | CyCYP719A42^PA,VI,SM^ | 0.0255 | 0.0223 | 0.0265 | 0.0248±0.0022 |
|  | CyCYP719A42^PA,VI,LD^ | 0.0378 | 0.0335 | 0.0337 | 0.0350±0.0024 |
|  | CyCYP719A42^SM,VI,LD^ | 0.0000 | 0.0000 | 0.0000 | 0.0000 |
|  | CyCYP719A42^PA,LD,SM^ | 0.0000 | 0.0000 | 0.0000 | 0.0000 |
|  | CyCYP719A42^PA,VI,SM,LD^ | 0.0000 | 0.0000 | 0.0000 | 0.0000 |
| (S)-cheilanthifoline to (S)-stylopine | Control | 0.0000 | 0.0000 | 0.0000 | 0.0000 |
|  | CyCYP719A39 | 0.0000 | 0.0000 | 0.0000 | 0.0000 |
|  | CyCYP719A39^MS^ | 0.0000 | 0.0000 | 0.0000 | 0.0000 |
|  | CyCYP719A39^IV^ | 0.0000 | 0.0000 | 0.0000 | 0.0000 |
|  | CyCYP719A39^PA^ | 0.0000 | 0.0000 | 0.0000 | 0.0000 |
|  | CyCYP719A39^DL^ | 0.0303 | 0.0365 | 0.0267 | 0.0312±0.0050 |
|  | CyCYP719A39^PA,DL^ | 0.0275 | 0.0276 | 0.0310 | 0.0287±0.0020 |
|  | CyCYP719A39^PA,MS^ | 0.0000 | 0.0000 | 0.0000 | 0.0000 |
|  | CyCYP719A39^PA,IV^ | 0.0000 | 0.0000 | 0.0000 | 0.0000 |
|  | CyCYP719A39^MS,IV^ | 0.0000 | 0.0000 | 0.0000 | 0.0000 |
|  | CyCYP719A39^MS,DL^ | 0.1116 | 0.0962 | 0.0977 | 0.1018±0.0085 |
|  | CyCYP719A39^IV,DL^ | 0.0000 | 0.0000 | 0.0000 | 0.0000 |
|  | CyCYP719A39^PA,IV,MS^ | 0.0000 | 0.0000 | 0.0000 | 0.0000 |
|  | CyCYP719A39^PA,IV,DL^ | 0.0000 | 0.0000 | 0.0000 | 0.0000 |
|  | CyCYP719A39^MS,IV,DL^ | 0.0385 | 0.0338 | 0.0352 | 0.0359±0.0024 |
|  | CyCYP719A39^PA,DL,MS^ | 0.0962 | 0.0860 | 0.0898 | 0.0906±0.0052 |
|  | CyCYP719A39^PA,IV,MS,DL^ | 0.0119 | 0.0143 | 0.0120 | 0.0128±0.0014 |
|  | CyCYP719A42 | 1.0000 | 1.0000 | 1.0000 | 1.0000 |
|  | CyCYP719A42^SM^ | 0.1056 | 0.0906 | 0.1010 | 0.0991±0.0077 |
|  | CyCYP719A42^VI^ | 0.7570 | 0.7169 | 0.7510 | 0.7417±0.0216 |
|  | CyCYP719A42^PA^ | 0.5620 | 0.5568 | 0.5774 | 0.5654±0.0107 |
|  | CyCYP719A42^LD^ | 0.0000 | 0.0000 | 0.0000 | 0.0000 |
|  | CyCYP719A42^PA,LD^ | 0.0000 | 0.0000 | 0.0000 | 0.0000 |
|  | CyCYP719A42^PA,SM^ | 0.1068 | 0.0931 | 0.0942 | 0.0980±0.0076 |
|  | CyCYP719A42^PA,VI^ | 0.4242 | 0.4033 | 0.4086 | 0.4121±0.0108 |
|  | CyCYP719A42^SM,VI^ | 0.0604 | 0.0588 | 0.0627 | 0.0607±0.0020 |
|  | CyCYP719A42^SM,LD^ | 0.0000 | 0.0000 | 0.0000 | 0.0000 |
|  | CyCYP719A42^VI,LD^ | 0.0000 | 0.0000 | 0.0000 | 0.0000 |
|  | CyCYP719A42^PA,VI,SM^ | 0.0241 | 0.0258 | 0.0249 | 0.0250±0.0009 |
|  | CyCYP719A42^PA,VI,LD^ | 0.0000 | 0.0000 | 0.0000 | 0.0000 |
|  | CyCYP719A42^SM,VI,LD^ | 0.0000 | 0.0000 | 0.0000 | 0.0000 |
|  | CyCYP719A42^PA,LD,SM^ | 0.0000 | 0.0000 | 0.0000 | 0.0000 |
|  | CyCYP719A42^PA,VI,SM,LD^ | 0.0000 | 0.0000 | 0.0000 | 0.0000 |
| (S)-nandinine to (S)-stylopine | Control | 0.0000 | 0.0000 | 0.0000 | 0.0000 |
|  | CyCYP719A39 | 1.0000 | 1.0000 | 1.0000 | 1.0000 |
|  | CyCYP719A39^MS^ | 1.2369 | 1.2552 | 1.2364 | 1.2428±0.0107 |
|  | CyCYP719A39^IV^ | 0.6088 | 0.5190 | 0.6030 | 0.5769±0.0503 |
|  | CyCYP719A39^PA^ | 0.6276 | 0.6134 | 0.6121 | 0.6177±0.0085 |
|  | CyCYP719A39^DL^ | 0.0000 | 0.0000 | 0.0000 | 0.0000 |
|  | CyCYP719A39^PA,DL^ | 0.0000 | 0.0000 | 0.0000 | 0.0000 |
|  | CyCYP719A39^PA,MS^ | 0.7482 | 0.7468 | 0.7449 | 0.7466±0.0016 |
|  | CyCYP719A39^PA,IV^ | 0.0000 | 0.0000 | 0.0000 | 0.0000 |
|  | CyCYP719A39^MS,IV^ | 0.7766 | 0.7573 | 0.8111 | 0.7816±0.0273 |
|  | CyCYP719A39^MS,DL^ | 0.0000 | 0.0000 | 0.0000 | 0.0000 |
|  | CyCYP719A39^IV,DL^ | 0.0000 | 0.0000 | 0.0000 | 0.0000 |
|  | CyCYP719A39^PA,IV,MS^ | 0.6445 | 0.6125 | 0.6117 | 0.6229±0.0187 |
|  | CyCYP719A39^PA,IV,DL^ | 0.0000 | 0.0000 | 0.0000 | 0.0000 |
|  | CyCYP719A39^MS,IV,DL^ | 0.0000 | 0.0000 | 0.0000 | 0.0000 |
|  | CyCYP719A39^PA,DL,MS^ | 0.0000 | 0.0000 | 0.0000 | 0.0000 |
|  | CyCYP719A39^PA,IV,MS,DL^ | 0.0000 | 0.0000 | 0.0000 | 0.0000 |
|  | CyCYP719A42 | 0.0000 | 0.0000 | 0.0000 | 0.0000 |
|  | CyCYP719A42 mutants | 0.0000 | 0.0000 | 0.0000 | 0.0000 |
| (S)-tetrahydrocolumbamine to (S)-tetrahydroberberine | Control | 0.0000 | 0.0000 | 0.0000 | 0.0000 |
|  | CyCYP719A42 | 1.0000 | 1.0000 | 1.0000 | 1.0000 |
|  | CyCYP719A42^SM^ | 0.0000 | 0.0000 | 0.0000 | 0.0000 |
|  | CyCYP719A42^VI^ | 0.2656 | 0.2592 | 0.2711 | 0.2653±0.0060 |
|  | CyCYP719A42^PA^ | 1.4961 | 1.5395 | 1.5035 | 1.5130±0.0232 |
|  | CyCYP719A42^LD^ | 0.0000 | 0.0000 | 0.0000 | 0.0000 |
|  | CyCYP719A42^PA,LD^ | 0.0000 | 0.0000 | 0.0000 | 0.0000 |
|  | CyCYP719A42^PA,SM^ | 0.0000 | 0.0000 | 0.0000 | 0.0000 |
|  | CyCYP719A42^PA,VI^ | 0.3003 | 0.3160 | 0.3348 | 0.3171±0.0173 |
|  | CyCYP719A42^SM,VI^ | 0.0000 | 0.0000 | 0.0000 | 0.0000 |
|  | CyCYP719A42^SM,LD^ | 0.0000 | 0.0000 | 0.0000 | 0.0000 |
|  | CyCYP719A42^VI,LD^ | 0.0000 | 0.0000 | 0.0000 | 0.0000 |
|  | CyCYP719A42^PA,VI,SM^ | 0.0000 | 0.0000 | 0.0000 | 0.0000 |
|  | CyCYP719A42^PA,VI,LD^ | 0.0000 | 0.0000 | 0.0000 | 0.0000 |
|  | CyCYP719A42^SM,VI,LD^ | 0.0000 | 0.0000 | 0.0000 | 0.0000 |
|  | CyCYP719A42^PA,LD,SM^ | 0.0000 | 0.0000 | 0.0000 | 0.0000 |
|  | CyCYP719A42^PA,VI,SM,LD^ | 0.0000 | 0.0000 | 0.0000 | 0.0000 |
|  | CyCYP719A39 | 0.0000 | 0.0000 | 0.0000 | 0.0000 |
|  | CyCYP719A39 mutants | 0.0000 | 0.0000 | 0.0000 | 0.0000 |

Table S5. Concentration of (*S*)-Stylopine produced by fermentation engineered strains

|  | Concentration (mg/L) | | | Mean value |
| --- | --- | --- | --- | --- |
| Control strain | 28.1678 | 30.7563 | 29.4101 | 29.44 |
| Mutant strain | 36.4854 | 33.5301 | 30.7014 | 33.57 |
